# Dynamic Lone Pairs and Fluoride-Ion Disorder in Cubic-BaSnF_4_

**DOI:** 10.1021/jacs.3c08232

**Published:** 2023-10-16

**Authors:** Briséïs Mercadier, Samuel W. Coles, Mathieu Duttine, Christophe Legein, Monique Body, Olaf J. Borkiewicz, Oleg Lebedev, Benjamin J. Morgan, Christian Masquelier, Damien Dambournet

**Affiliations:** † Réseau sur le Stockage Electrochimique de l’Energie, RS2E, FR CNRS 3459, 80039 Amiens Cedex, France; ‡ Sorbonne Université, CNRS, Physicochimie des Electrolytes et Nanosystèmes Interfaciaux, UMR CNRS 8234, 75005 Paris, France; § Laboratoire de Réactivité et de Chimie du Solides, UMR CNRS 7314, 80039 Amiens Cedex, France; ∥ Department of Chemistry, University of Bath, Claverton Down, Bath BA2 7AY, United Kingdom; ⊥ Quad One, Harwell Science and Innovation Campus, The Faraday Institution, Didcot OX11 0RA, United Kingdom; # Institut de Chimie de la Matière Condensée de Bordeaux, UMR CNRS 5026, 33608 Pessac, France; ∇ Institut des Molécules et Matériaux du Mans, UMR CNRS 6283, Le Mans Université, 72085 Le Mans Cedex 9, France; ○ X-ray Science Division, Advanced Photon Source, Argonne National Laboratory, Lemont, Illinois 60439, United States; ◆ Laboratoire de Cristallographie et Sciences des Matériaux, CRISMAT, 14000 Caen, France

## Abstract

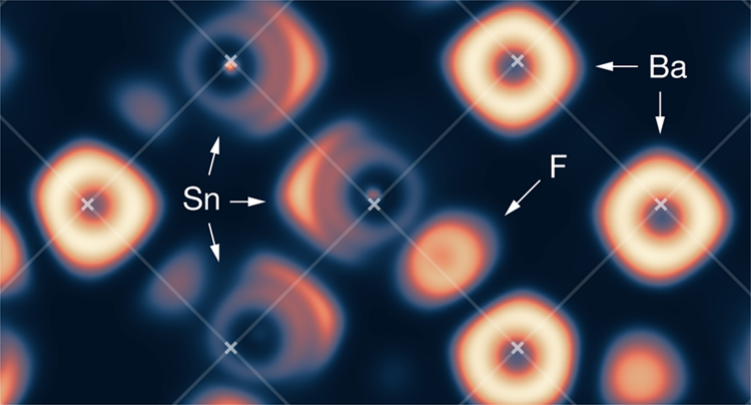

Introducing compositional or structural disorder within crystalline solid electrolytes is a common strategy for increasing their ionic conductivity. (M,Sn)F_2_ fluorites have previously been proposed to exhibit two forms of disorder within their cationic host frameworks: occupational disorder from randomly distributed M and Sn cations and orientational disorder from Sn(II) stereoactive lone pairs. Here, we characterize the structure and fluoride-ion dynamics of cubic BaSnF_4_, using a combination of experimental and computational techniques. Rietveld refinement of the X-ray diffraction (XRD) data confirms an average fluorite structure with {Ba,Sn} cation disorder, and the ^119^Sn Mössbauer spectrum demonstrates the presence of stereoactive Sn(II) lone pairs. X-ray total-scattering PDF analysis and *ab initio* molecular dynamics simulations reveal a complex local structure with a high degree of intrinsic fluoride-ion disorder, where 1/3 of fluoride ions occupy octahedral “interstitial” sites: this fluoride-ion disorder is a consequence of repulsion between Sn lone pairs and fluoride ions that destabilizes Sn-coordinated tetrahedral fluoride-ion sites. Variable-temperature ^19^F NMR experiments and analysis of our molecular dynamics simulations reveal highly inhomogeneous fluoride-ion dynamics, with fluoride ions in Sn-rich local environments significantly more mobile than those in Ba-rich environments. Our simulations also reveal dynamical reorientation of the Sn lone pairs that is biased by the local cation configuration and coupled to the local fluoride-ion dynamics. We end by discussing the effect of host-framework disorder on long-range diffusion pathways in cubic BaSnF_4_.

## Introduction

The ability of solid electrolytes to allow ion transport in the solid state makes them useful for a range of applications, including fuel cells and solid-state batteries.^1−5^ As a consequence, a considerable amount of research has focused on understanding how the structure and chemical composition of particular solid electrolytes modulate their ionic conductivity.^3,6−13^ In addition to providing insight into the atomic-scale mechanisms of ionic conductivity within specific families of solid electrolytes, this body of research has also produced various “design principles”—general conceptual models that seek to explain, and predict, trends in ionic conductivity across different families of solid electrolytes.^8,12,14−19^ Many of these solid electrolyte design principles reflect how changes to the structure and composition of the host framework—the set of nondiffusive ions within a solid electrolyte—modulate the potential energy surface for the mobile ions, and hence influence overall ionic conductivity.^6,11,16,19−21^

One such design principle arises from the observation that solid electrolytes with some form of host-framework disorder often have significantly higher ionic conductivities than related compounds with well-ordered host frameworks.^10,15,22−30^ Framework-disordered solid electrolytes typically exhibit one of two classes of disorder: occupational disorder, where two or more distinct species occupy the same crystallographic positions,^10,15,24,28,29,31−36^ or orientational disorder, where molecular or polyatomic subunits within the host framework have different disordered orientations.^37−40^ Orientational disorder can be static, where each polyatomic subunit has a fixed average orientation over experimentally relevant time scales,^41,42^ or dynamic, where the polyatomic subunits rotate and reorient.^40,43,44^ In some solid electrolytes, this reorientational dynamics of the host framework is thought to couple to the diffusive dynamics of the mobile-ion species, giving rise to a so-called “paddlewheel” effect.^21,38,43,45^

While orientational disorder in solid electrolytes is usually discussed in the context of molecular or polyanion orientational degrees of freedom, materials that contain post-transition metals with “stereoactive” lone pairs, such as Sn or Bi, may exhibit electronic orientational disorder.^46^ These cations, when in an oxidation state two fewer than their formal maximum (e.g., Sn^II^ or Ba^II^), have a formal electron configuration with a filled s-orbital as their last valence shell. These s^2^ states can mix with neighboring-anion p states to form a bonding state and an antibonding state, and this antibonding state mixes with metal p states to form an asymmetric lone-pair state, characterized by an eccentric (off-center with respect to the atomic nucleus) “stereoactive” charge density, that directs the cation coordination geometry and often results in distorted low-symmetry cation coordination environments.^47,48^

In many materials that contain stereoactive lone pairs, the local distortions due to these lone pairs are correlated over long length scales. These materials are long-range ordered, and their structures can be determined using average crystallographic techniques such as Bragg diffraction. In other materials, however, the distortions due to stereoactive lone pairs are uncorrelated.^46,49,50^ These materials are crystallographically disordered, and average-structure crystallographic methods yield inaccurate high-symmetry structural models. Because the distortion around each cation depends on the relative orientation of the corresponding lone pair, this behavior can be considered a form of orientational disorder, analogous to molecular or polyanionic orientational disorder as discussed above. These lone-pair effects may also be dynamic, showing fluctations^46,51−53^ or even rotations^54,55^ of the lone-pair charge density, mirroring the dynamic orientational disorder of “paddlewheel” materials.^21,38,45^

While the role of stereoactive lone pairs in solid electrolytes has previously been discussed in the context of crystallographically ordered materials,^56−60^ a detailed understanding of lone-pair behavior and its effect on ion transport in crystallographically disordered solid electrolytes is currently lacking.^61−63^

Here, we report a combined experimental and computational study of the fluorite-structured fluoride-ion conductor cubic (c-)BaSnF_4_. We find that c-BaSnF_4_ exhibits both site-occupational disorder due to Ba/Sn cation mixing and dynamic orientational disorder due to Sn stereoactive lone pairs. The combination of these two forms of host-framework disorder within an ion-conducting material makes c-BaSnF_4_ a particularly interesting focus of study. We show that these two forms of disorder are coupled and that together they strongly influence the structure and dynamics of the mobile fluoride ions. The fluoride-ion substructure is highly disordered, with 1/3 of fluoride ions occupying “interstitial” sites, due to lone-pair repulsion of fluoride ions in highly tin-coordinated sites. Fluoride-ion dynamics are strongly dependent on the local cation environment and are coupled to dynamical reorientations of neighboring Sn lone pairs. Our study provides new insight into the rich structural and dynamical behavior of fluoride-ion-conducting c-BaSnF_4_ and how this arises from the unusual combination of coupled site-occupation and lone-pair orientation disorder within the host framework.

### Cation Disorder and Stereoactive Lone Pairs in F-Ion-Conducting Fluorites

The study of fluoride-ion-conducting fluorites has a long history, starting from Faraday’s discovery of superionic β-PbF_2_.^64^ More recently, this family of materials have been the subject of renewed interest because of their potential use as electrolytes in solid-state fluoride-ion batteries.^65−68^

**Figure 1:**
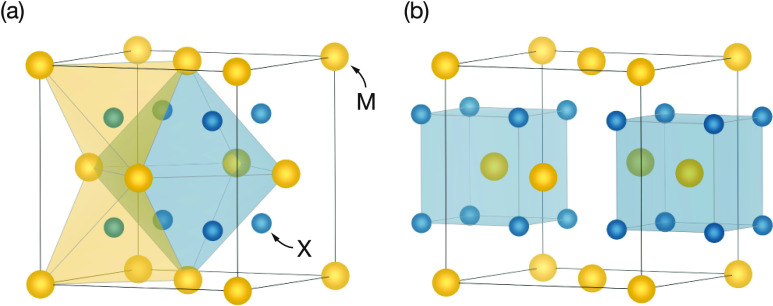
Schematic of the MX_2_ fluorite structure (space group *Fm*3̅*m*), highlighting (a) tetrahedral (normal; Wyckoff 8c) and octahedral (interstitial; Wyckoff 4b) anion sites and (b) cubic cation sites (Wyckoff 4a).

The fluorite structure is composed of a face-centered-cubic cation lattice, with anions occupying all of the tetrahedral holes (Figure 1a). The octahedral holes are vacant and are usually considered as “interstitial” sites. An alternative structural description is obtained by considering the positions of cations within an anionic substructure (Figure 1b): from this perspective, the anions define a simple-cubic lattice and the cations occupy half the cubic holes, giving 8-fold MX_8_ cation coordination.

In conventional fluorites, such as CaF_2_, anion transport requires the presence of thermally generated point defects—vacancies and interstitials—within the anionic substructure.^69−72^ Fluorites are typically anti-Frenkel disordered: some fraction of anions occupy octahedral interstitial sites, leaving an equal number of tetrahedral sites vacant.^69,73^ While additional interstitials and vacancies can be introduced by aliovalent doping, the intrinsic defect concentration depends on the ease with which anti-Frenkel pairs can form, which, in turn, is approximately dependent on the relative energies of ions occupying the tetrahedral and octahedral anion sites within the fcc cationic host framework. In simple fluorites, the anti-Frenkel-pair formation energy is usually high: for CaF_2_, anion Frenkel-pair formation energies have been calculated as 2.2–2.9 eV.^69,74−76^ As a consequence, at low-to-moderate temperatures, these materials have low fluoride-ion defect concentrations and corresponding low ionic conductivities.^56,77^

Significantly higher ionic conductivities are found in fluorite-structured materials that contain cations with stereoactive lone pairs, such as β-PbF_2_.^56,77,78^ This effect has been suggested to be a possible consequence of the high polarizability of the cation facilitating diffusion of the mobile anions,^56,78,79^ or of the negative charge of the Pb lone pairs electrostatically destabilizing adjacent fluoride ions in tetrahedral sites, thereby promoting the formation of anti-Frenkel pairs.^56^ Neutron diffraction data^80^ and AIMD simulations^81^ of β-PbF_2_, however, show no evidence for octahedral site occupation by fluoride ions, bringing into question the hypothesis that the presence of stereoactive lone pairs in fluorite-structured materials promotes Frenkel-pair formation.^82^

A second class of fluorite-structured materials that exhibit particularly high ionic conductivities are disordered mixed-cation systems, M_x_^′^M_1–*x*_^″^F_2_.^24,33,78,83−86^ The highest ionic conductivity materials in this class are those with different valence cations, such as RbBiF_4_, where cation disorder induces high levels of anion disorder.^84,87^ A significant increase in ionic conductivity compared to analogous single-cation fluorites is also observed for mixed-cation systems where both cations have a formal 2+ oxidation state, such as Ba_1–*x*_Ca_1+*x*_F_2_:^24,25,33^ the presence of cation disorder again causes considerable fluoride disorder with fluoride ions displaced significantly from their ideal crystallographic positions to form “pseudovacancies”.^24^

Some fluorite materials exhibit both stereoactive lone pairs and cation mixing.^62,87−90^ Of particular relevance to the present study is the work of Dénès et al. on Ca_1–*x*_Sn_*x*_F_2_ (*x* = 0.27).^62,88^ For this system, X-ray diffraction (XRD) data give a cubic fluorite average structure, consistent with a solid solution of Ca and Sn distributed randomly over the cation positions, and ^119^Sn Mössbauer data show a large quadrupole doublet, characteristic of a stereoactive tin lone pair.

The presence of a stereoactive tin lone pair requires an asymmetric tin coordination environment, which is inconsistent with the structural model implied by the diffraction data, in which Ca and Sn both have cubic MF_8_ coordination (*cf.*Figure 1b). To reconcile these apparently contradictory data, Dénès and co-workers proposed a structural model wherein each tin is displaced toward one face of the enclosing [F8] cube to give square-pyramidal SnF_4_E coordination, where E denotes the stereoactive lone pair, with this lone pair oriented toward the more distant [F8] cube-face. These tin lone pairs are assumed to orient randomly along each possible ⟨100⟩ direction to give an average structure with cubic symmetry, consistent with the experimental diffraction data.

The structural model proposed by Dénès et al. implies that Ca_1–*x*_Sn_*x*_F_2_ exhibits both occupational cation disorder and Sn lone-pair orientational disorder, and similar behavior might be expected in other mixed-cation fluorites where one cation species has a stereoactive lone pair.^89,90^ Such mixed-cation fluorites are interesting to examine in the context of understanding how these distinct but coexisting forms of host-framework disorder together modulate the structure and dynamics of the mobile anion species. Here, we focus on the structure and fluoride-ion dynamics of c-BaSnF_4_, which we consider to be a representative member of this family of mixed-cation fluorites. c-BaSnF_4_ is also of practical interest due to its shared composition with the more widely studied layered tetragonal phase t-BaSnF_4_,^60,91,92^ which is considered to be a prospective solid electrolyte for fluoride-ion batteries.^93,94^

## Materials and Methods

### Synthesis

Cubic BaSnF_4_ was synthesized via a ball-milling process using a planetary mill (Fritsch Pulverisette 6). Precursors (SnF_2_, Sigma-Aldrich, 99%; BaF_2_, Sigma-Aldrich 99.99%) were dried at 150 °C under vacuum for 3 h and stored under Ar inert atmosphere. The desired amounts of precursors were weighed and sealed in Zirconia milling jars in an argon-filled glovebox, with a powder-to-ball ratio of 1:13. The balls were 10 mm in diameter and made out of zirconia. The precursors were then milled at 400 rotations/min for 12 h, divided into 24 cycles. Each cycle consisted of 15 min of milling and 15 min of pause, which prevented overheating.

### Impedance Spectroscopy

Electrochemical Impedance spectroscopy was performed on c-BaSnF_4_ powder pressed into a pellet. Gold was sputtered on both sides of the pellet to guarantee good contact. A BioLogic MTZ-35 impedance analyzer was used to collect data in a frequency range of 3.5 × 10^7^ Hz to 1 Hz, under an Ar atmosphere. The resulting data were fitted using the equivalent circuit model proposed in ref (95).

### X-ray Diffraction

X-ray diffraction was performed using a Bruker D8 Advance powder diffractometer with a copper anode (Cu Kα = 1.54059 Å). The powder XRD pattern was fitted using the Rietveld method as implemented in the Fullprof program,^96^ with a split pseudo-Voigt function used to model the peaks.^97^

### ^119^Sn Mössbauer Spectroscopy

A lab-made constant acceleration Halder-type spectrometer operating in transmission geometry was used to carry out the Mössbauer analyses. The spectrometer was equipped with a radioactive source of ^119m^Sn (370 MBq) embedded in a CaSnO_3_ matrix and maintained at room temperature. Experiments were performed with 50–70 mg of sample ([Sn] = 5–8 mg cm^–2^) at room temperature (∼293 K) and 77 K using a liquid nitrogen bath cryostat. The Mössbauer hyperfine parameters (δ isomer shift, Δ quadrupole splitting, Γ signal line width, *G*_11_ Goldanskii–Karyagin factor, and relative areas) were refined using the WinNormos software.^98^ Isomer shift values are reported relative to those of CaSnO_3_ at room temperature.

### ^19^F Solid-State NMR

Quantitative ^19^F Magic Angle Spinning (MAS) NMR spectra were recorded on Bruker Avance III spectrometers operating at *B*_0_ = 7.0 T (^19^F Larmor frequency of 282.4 mHz), using a 1.3 mm CP-MAS probe head, and, for variable-temperature experiments, using a 2.5 mm double-resonance (^1^H/^19^F–X) CP-MAS probe and a Bruker Cooling Unit (BCU-II). The ^19^F MAS spectra were recorded by using a Hahn echo sequence with an interpulse delay equal to one rotor period. The 90° pulse lengths were set to 1.25 μs (for SnF_2_ and BaSnF_4_) and 1.5 μs (BaF_2_) and the recycle delays were set to 900 s (for SnF_2_) and 300 s (for BaF_2_ and BaSnF_4_) using the 1.3 mm CP-MAS probe head. For the variable-temperature experiments, using the 2.5 mm CP-MAS probe head, the 90° pulse length was set to 2 μs and the recycle delay was set to 10 s. The temperature inside the rotor was estimated from the chemical shift and spin–lattice relaxation time (*T*_1_) of ^79^Br in KBr powder.^99^^19^F spectra are referenced to CFCl_3_ and were fitted using the dmfit software.^100^

### Pair-Distribution Functions

Pair-distribution function (PDF) measurements were performed at the 11-ID-B beamline at the Advanced Photon Source at the Argonne National Laboratory. High-energy synchrotron XRD (λ = 0.2128 Å) 2D total-scattering data was collected and integrated into one-dimensional diffraction data using FIT2D.^101^ The PDFgetX3 software was used to carry out Fourier transformation and correction of the PDFs.^102^ Refinements were performed using the software PDFgui.^103^

### Molecular Dynamics Simulations

To model the equilibrium structure and dynamics of c-BaSnF_4_, we performed *ab initio* molecular dynamics (AIMD) using the Vienna *ab initio* simulation package (VASP).^104−106^ We used the revised Perdew–Burke–Ernzerhof generalized gradient approximation PBEsol exchange–correlation function.^107^ Interactions between core and valence electrons were described within the projector-augmented-wave (PAW) method,^108^ with cores of [Kr] 4d^10^ for Ba, [Kr] for Sn, and [He] for F. We simulated a 6 × 6 × 6 supercell, starting from a cation-disordered fluorite structure with a special quasi-random configuration of Ba and Sn over the Wyckoff 4a cation sites, generated using the icet package.^109^ This 6 × 6 × 6 special quasi-random structure best approximates the Ba/Sn correlations for an infinite lattice with a fully random arrangement of cations.^110,111^

Our molecular dynamics simulation used a plane-wave cutoff of 350 eV with only the Γ point used for *k*-space sampling and without spin-polarization. The simulation was performed at 600 K and used a time step of 2 fs. Before our production run, we obtained the 600 K equilibrium volume by running a preliminary series of simulations with different cell volumes for 8 ps each, and fitting the Birch–Murnaghan equation to the resulting energy–volume data set. The simulation was run in the *NVT* ensemble by using a Nosé–Hoover thermostat. Thermal equilibration was performed by running a 2 ps *NVE* run with the temperature rescaling every 50 steps. The production run was 159 ps in length.

To calculate fluoride-ion site occupancies and site–site transition frequencies, at each simulation time step we assigned every fluoride ion to a distinct tetrahedral or octahedral site by projecting the instantaneous fluoride-ion positions onto polyhedral “sites” defined by the Wyckoff 4a positions as fixed vertex positions (1).

For structural analysis (calculation of radial distribution functions and cation–4a displacements), we extracted a set of “inherent” structures^112−114^ from our simulation trajectory by performing a conjugate-gradient geometry optimization on configurations selected every 50 timesteps. Each inherent structure represents a local minimum on the corresponding 3*N*-dimensional potential energy surface. To calculate an example electron localization function (ELF),^115^ we performed full geometry optimizations with a cutoff of 500 eV with a minimum *k*-point spacing of 0.25 Å^–1^, with atomic positions and cell parameters relaxed until all atomic forces were less than 2 × 10^–2^ eV Å^–1^.

To obtain tin lone-pair orientations, we calculated the set of maximally localized Wannier functions^116^ for structures sampled every 50 ps, using the Wannier90 code.^117^ The net dipole on each tin atom was calculated by associating each Wannier center with the closest ion, and then, for each tin, summing over all associated Wannier-center displacement vectors.^118^ We assume that tin polarization is dominated by contributions from the lone-pair states and that our calculated polarization vectors therefore characterize these lone-pair orientations.

Analysis of the simulation data was performed using the RevelsMD,^119^ Site-Analysis,^120^ ASE,^121^ Pymatgen,^122^ Numpy,^123^ and Scipy^124^ codes. The time-average fluorine density (Figure 6) was calculated using a linear combination of a conventional histogram with a triangular kernel and a force-extrapolated analogue, as described in refs (125−127).

## Results and Discussion

### Structural Characterization

**Figure 2:**
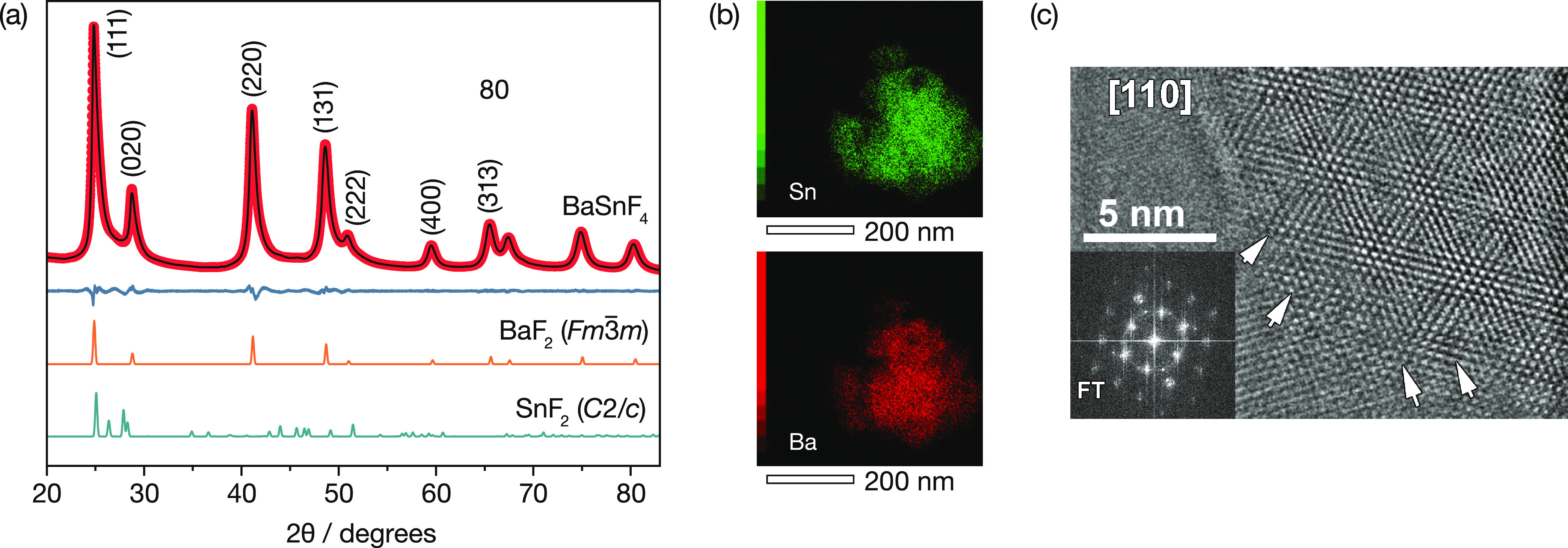
(a) Powder XRD pattern and Rietveld analysis of c-BaSnF_4_. Reference patterns of the BaF_2_ and SnF_2_ precursors are presented at the bottom. (b) Distribution of tin (green; top) and barium (red; bottom) within a c-BaSnF_4_ particle, seen via EDX mapping. (c) HRTEM image of c-BaSnF_4_.

Cubic BaSnF_4_ was synthesized by mechanically milling BaF_2_ and SnF_2_ in a manner similar to ref (95) (see the Materials and Methods section). The X-ray diffraction pattern (Figure 2) indexes to a face-centered-cubic structure from the *Fm*3̅*m* (225) space group, consistent with an average fluorite structure. The X-ray pattern shows no visible peaks for the parent SnF_2_ (*C*2/*c*) phase, and energy-dispersive X-ray analysis (EDX mapping) shows homogeneous distributions for both Sn and Ba. Quantitative analysis of the EDX mapping data gives proportions of Ba and Sn of 49.6(7) and 50.3(7)%, respectively, which is close to the nominal 1:1 Ba/Sn stoichiometry (see Figure S2 for the full mapping data). As a further check on the synthesized compound, we performed electrochemical impedance spectroscopy, and obtained an ionic conductivity at 30 °C of 4.6 × 10^–6^ cm^–1^, with an activation energy of 0.56 eV. This ionic conductivity is consistent with previous literature values for c-BaSnF_4_,^95^ and is >10^3^ higher than that of fluorite-structured BaF_2_,^24^ illustrating the positive effect of cation mixing on fluoride-ion transport.

Our XRD data show no superstructure reflections, indicating that Ba and Sn are disordered over the cation sites. From indexing the XRD data, we obtain a cell parameter of *a* = 6.1945(2) Å, which is close to the value for pristine BaF_2_ of *a* = 6.1964(2) Å.^128^ This result is somewhat unexpected, given the smaller ionic radius of Sn^2+^ compared to Ba^2+^, and suggests the possibility of local distortions in the cation substructure. Düvel et al. reported similar excess-volume behavior in Ba_1–*x*_Ca_*x*_F_2_ solid solutions,^24^ where this was proposed as a contributing factor to enhanced fluoride-ion transport relative to the end-members. HRTEM data provide further evidence of local deviations from an ideal fluorite-type structure; these show visible changes in inter-reticular distances (Figure 2c, white arrows) that indicate regions of local strain.

**Figure 3:**
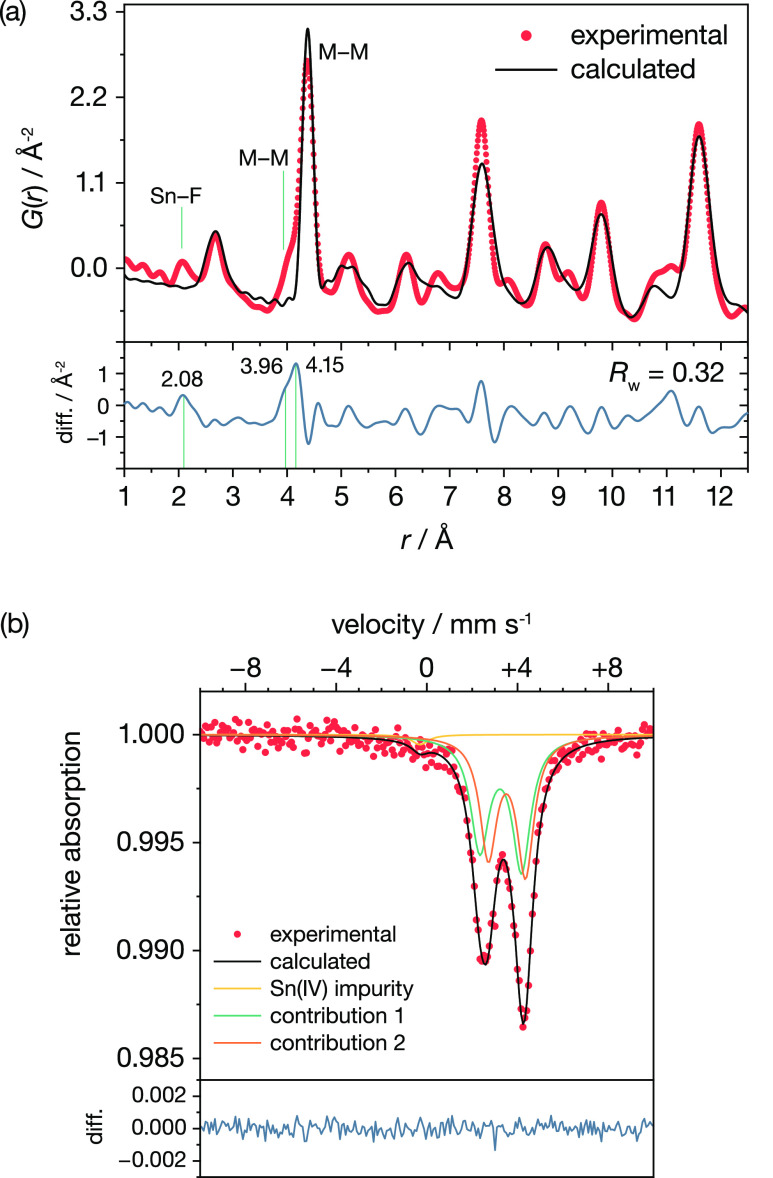
(a) PDF refinement obtained by using a cubic model. The main atomic distances referred to in the main text have been labeled. The blue curve shows the difference between calculated and experimental data. (b) Room-temperature (293 K) ^119^Sn Mössbauer spectrum of c-BaSnF_4_. Fitting parameters for the two contributions shown are provided in the Supporting Information.

Additional structural information is given by our X-ray total-scattering PDF data. For interatomic distances between 12 and 50 Å, the PDF data are relatively well described by a cubic fluorite *Fm*3̅*m* model (*R*_w_ = 20%; see Figure S3). At short range, however (between 1 and 12 Å; Figure 3a), this high-symmetry structural model gives a poor fit to the PDF data (*R*_w_ = 32%), indicating that the local structure of c-BaSnF_4_ deviates significantly from an ideal fluorite-type structure. The cubic model fails to predict the peak observed at *r* = 2.08 Å and the apparent splitting at *r* = 3.96–4.15 Å. In other fluorides, Sn adopts short Sn–F distances (e.g., 2.28 Å in tetragonal BaSnF_4_,^95^ and as short as 2.03 Å in SnF_2_^129^). We therefore provisionally assign the peak at *r* = 2.08 Å to short Sn–F bonds, which requires that Sn or F, or both species, are displaced from their ideal fluorite positions.

Figure 3b shows the room-temperature ^119^Sn Mössbauer spectrum for our c-BaSnF_4_ sample. The spectrum features an asymmetric quadrupole doublet with an isomer shift of around 3 mm s^–1^, characteristic of covalently bonded Sn(II), and a large quadrupole splitting parameter (Δ > 1.5 mm s^–1^), indicating that Sn exhibits a stereoactive lone pair.^62,130^ The experimental spectrum can be reconstructed using two quadrupole doublets with distinct isomer shift and quadrupole-splitting parameters (see the Supporting Information for details), indicating some degree of variation in Sn–F bonding interactions and in the coordination geometry around individual tin cations.

Dénès and co-workers have previously proposed a structural model for fluorite Ca_1–*x*_Sn_*x*_F_2_, on the basis of experimental XRD and Mössbauer data similar to those reported here.^62,88^ In that model, the presence of a tin stereoactive lone pair causes each tin cation to be displaced toward one face of its enclosing [F8] cube, giving square-pyramidal SnF_4_E coordination with a reduced nearest-neighbor Sn–F distance. This structural model at first appears to be consistent with our XRD and Mössbauer data and, hence, to provide an explanation for the deviation from the ideal fluorite structure evident in the short-range PDF data described above. The position of the first peak in our PDF data, however, at *r* = 2.08 Å, is too short to be explained by square-pyramidal SnF_4_E coordination within an ideal cubic array of fluoride ions: the shortest possible Sn–F distance from this model is *a*√2/2 = 2.19 Å. We therefore interpret the PDF feature at *r* = 2.08 Å as indicative of a significant degree of distortion to the fluorine substructure away from the reference simple-cubic structure. The structural model of Dénès et al. also predicts equivalent SnF_4_E coordination for all tin cations, which is inconsistent with the apparent variation in bonding and coordination geometry for tin cations evidenced by the Mössbauer data.

**Figure 4:**
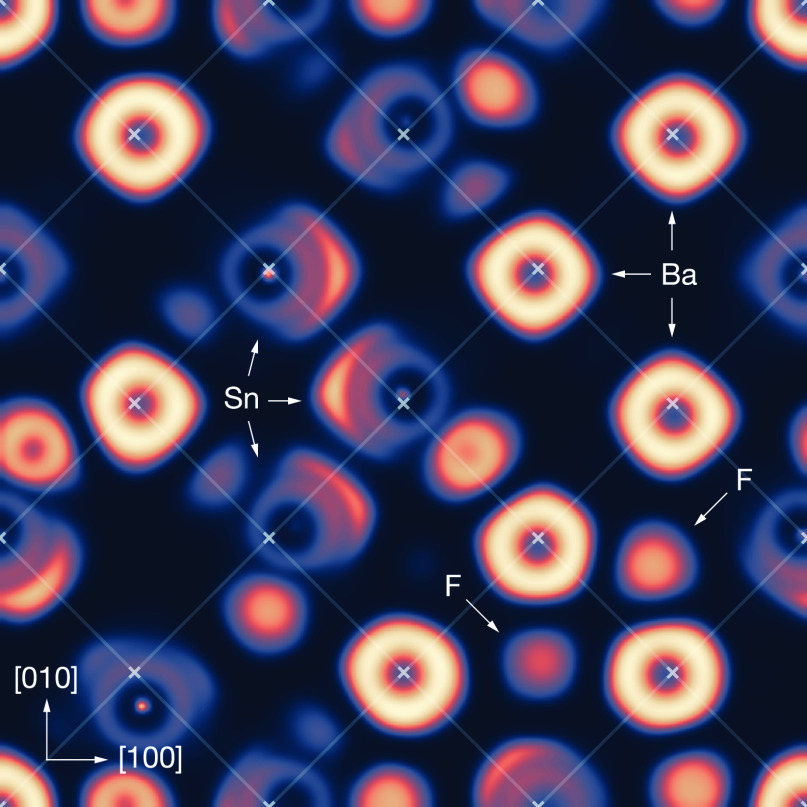
(001) Cross section through the electron localization function calculated for c-BaSnF_4_ for a single structure quenched from AIMD simulations.^115^ Crosses show ideal cation positions (*Fm*3̅*m* Wyckoff 4a) for a fluorite (*Fm*3̅*m*) structure.

More detail about the local structure of c-BaSnF_4_, including the behavior of the Sn lone pair, is provided by analyzing structures obtained by quenching from an AIMD simulation. Figure 4 shows a (001) cross section through the electron localization function (ELF),^115^ calculated for a quenched structure from our AIMD simulation. This cross section intersects with the Wyckoff 4a positions that are occupied by cations in the perfect fluoride structure. Atoms are visible as regions of nonzero ELF density, and each chemical species, Ba, Sn, and F, has a distinct appearance. Ba atoms are visible as bright symmetric rings that are centered approximately on the 4a positions, indicating that barium sits close to its ideal fluorite position. Sn atoms appears as less bright rings, with bright eccentric lobes that correspond to stereoactive lone pairs. These lone pairs are generally oriented approximately along ⟨100⟩ directions. The Sn centers appear either to be close to the 4a positions or, where they are displaced, the displacement appears uncorrelated with the orientation of the lone pair.

We also observe ELF features due to fluoride ions, even though the (001) plane in the figure contains no tetrahedral 8c sites and therefore should contain no fluoride ions for a perfect fluoride structure. However, we observe a number of fluoride ions occupying either octahedral or tetrahedral-edge positions, showing a high degree of fluoride-ion disorder.

**Figure 5:**
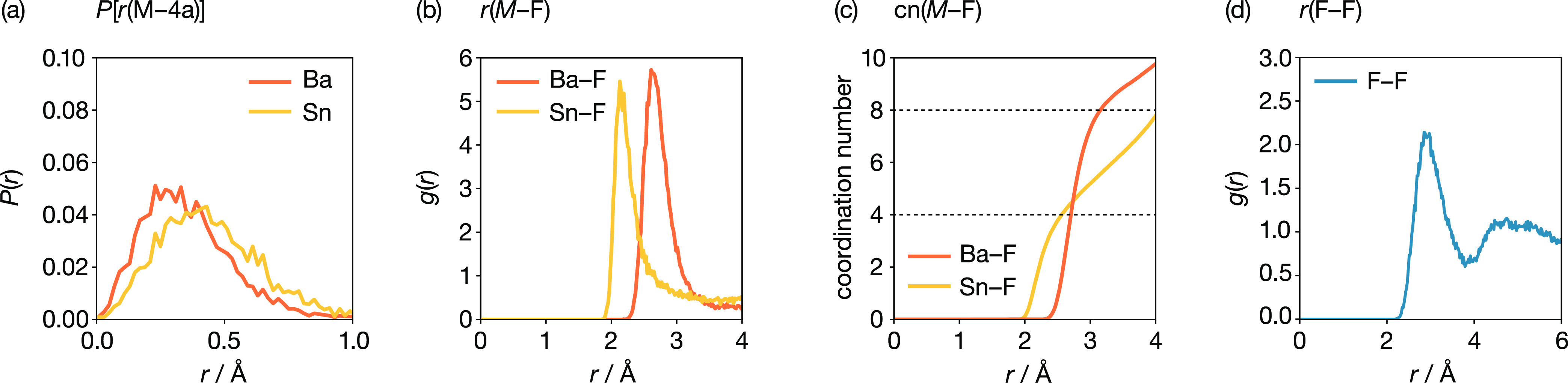
(a) Probability density functions *P*(*r*)– between Ba and Sn and their respective nearest *Fm*3̅*m* Wyckoff 4a positions. (b) M–F radial distribution functions (M = {Sn, Ba}). (c) M–F coordination number functions (M = {Sn, Ba}). The dashed horizontal lines indicate coordination numbers of 4 and 8. (d) F–F radial distribution function.

A more quantitative analysis of the c-BaSnF_4_ structure is presented in Figure 5. Figure 5a shows the probability distributions of cation distances from their closest 4a site, *P*[*r*(*M*–4a)], for Ba and Sn. Both cation species are, on average, displaced from their corresponding ideal fluorite cation positions, indicating how the cation substructure is locally distorted from a perfect fcc lattice. On average, Sn is displaced further from the nearest 4a position than Ba, which is consistent with the smaller size of Sn. In general, however, the two probability distribution functions have similar shapes, indicating no qualitative difference between the Ba and Sn positions relative to their formal crystallographic sites.

Figure 5b shows the Ba–F and Sn–F radial distribution functions (RDFs), *g*(*r*). The nearest-neighbor M–F distance is shorter for Sn (∼2.1 Å) than for Ba (∼2.7 Å), and confirms our earlier assignment of the feature at 2.08 Å in our PDF data (Figure 3b). Integrating these RDFs gives cumulative coordination numbers, which are shown in Figure 5c. The coordination number for Ba rises sharply to ∼8, which is the expected number for fluorite-like MF_8_ cation coordination. The coordination number for Sn, however, initially only rises to ∼4, indicating that, on average, each Sn has only four neighboring fluoride ions.

Dénès et al. have previously proposed that in Ca_*x*_Sn_1–*x*_F_2_, Sn is displaced from the ideal fluorite 4a position toward one face of an enclosing [F8] cube, to accommodate the stereoactive Sn lone pair.^62,88^ For c-BaSnF_4_, if Sn were sitting off-center in a well-formed [F8] cube, then the coordination number would show two distinct steps corresponding to [4 + 4] coordination. The calculated Sn–F coordination number, however, rises continuously after the first step, reaching ∼8 at 4 Å: the average coordination environment around Sn includes four neighboring F that occupy 8c-type positions, with four more F in some diffuse disordered arrangement at distances of 2.3–4.0 Å. Our data therefore suggest an alternative model for the Sn-coordination environment, where the Sn lone pair is accommodated not by Sn being displaced significantly from the 4a position, but instead by a significant disruption of the fluoride ions on the lone-pair-adjacent face of the nominal [F8] coordination environment, which presumably reduces the mutual electrostatic repulsion between these fluoride ions and the proximate Sn lone pair.^131^ Additional evidence for significant disordering of fluoride ions comes from the F–F RDF (Figure 5d), which shows a very weak second peak, more typical of an amorphous glassy phase than a regular crystalline array of atoms.

**Figure 6:**
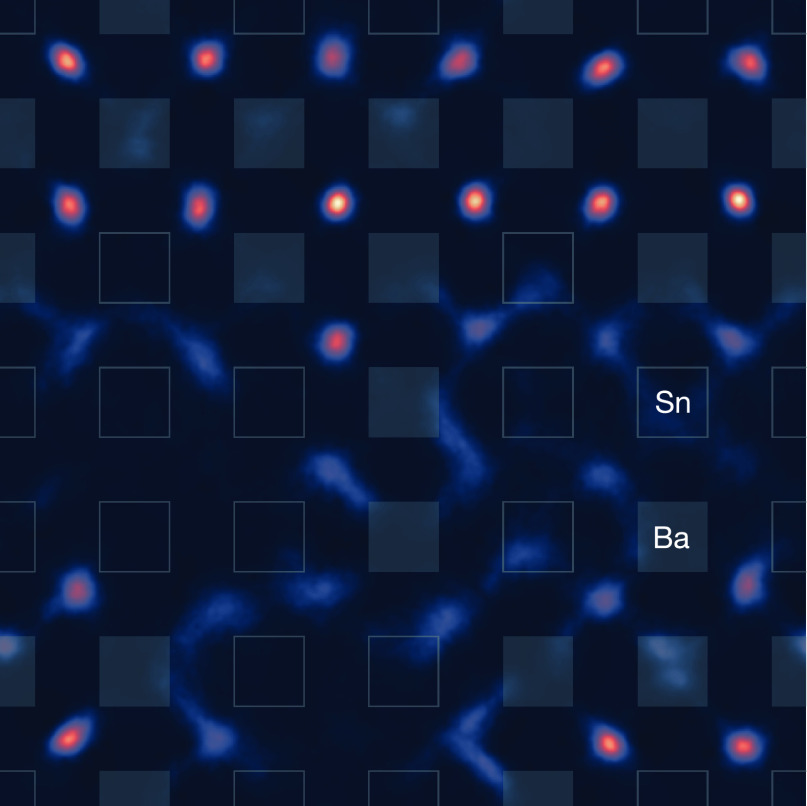
(001)-Projected slice through the time-average fluoride-ion density of c-BaSnF_4_ from the AIMD simulation at 600 K, showing a single plane of tetrahedral sites. Each tetrahedral site is coordinated by four cations (*cf.*, Figure 1a), which have their projected positions marked by squares. Filled squares indicate Ba positions, and empty squares indicate Sn positions.

Figure 6 shows a section through the time-average fluoride-ion density calculated from our AIMD simulation. The section is centered on a (001) plane of tetrahedral 8c positions. The superimposed closed and open squares indicate the Ba and Sn atoms, respectively, that tetrahedrally coordinate these 8c positions. The fluoride-ion density is highly heterogeneous and shows stark qualitative differences between Ba-rich regions and Sn-rich regions. In Ba-rich regions, the fluoride-ion density is well localized around the 8c positions, as expected for a conventional fluorite structure. In Sn-rich regions, however, the fluoride-ion density is highly diffuse, which is consistent with the proposal above that Sn lone pairs are associated with significant disorder in the local fluoride substructure. These fluoride-ion–density data also suggest that the dynamic behavior of the fluoride ions is strongly dependent on the identity of the nearby cation species: fluoride ions in Ba-rich regions of c-BaSnF_4_ appear to be relatively immobile, while fluoride ions in Sn-rich regions appear to be much more mobile, and we return to this point below.

To obtain another perspective on the degree of fluoride-ion disorder, we project the instantaneous fluoride-ion positions from our AIMD simulation trajectory onto discrete tetrahedral or octahedral sites defined by the set of Wyckoff 4a sites that define their vertices. This site projection gives a noteable 1/3 of fluoride ions occupying octahedral “interstitial” sites rather than conventional tetrahedral sites—i.e., individual octahedral sites are, on average, equally likely to be occupied by fluoride ions than individual tetrahedral sites. This degree of fluoride-ion site disorder is even greater than the “massive disorder” found in RbBiF_4_,^132^ where 1/4 of fluoride ions occupy nominally octahedral positions.^133^ Furthermore, this disorder is not simply a large number of thermally generated anion “Frenkel pairs”: quenching from our AIMD simulation produces a 0 K structure with this same proportion of fluoride ions occupying octahedral sites that is 16.7 meV/atom lower in energy than the corresponding optimized structure with all fluoride ions occupying tetrahedral positions. This extreme fluoride-ion disorder is therefore *intrinsic* to c-BaSnF_4_.

**Figure 7:**
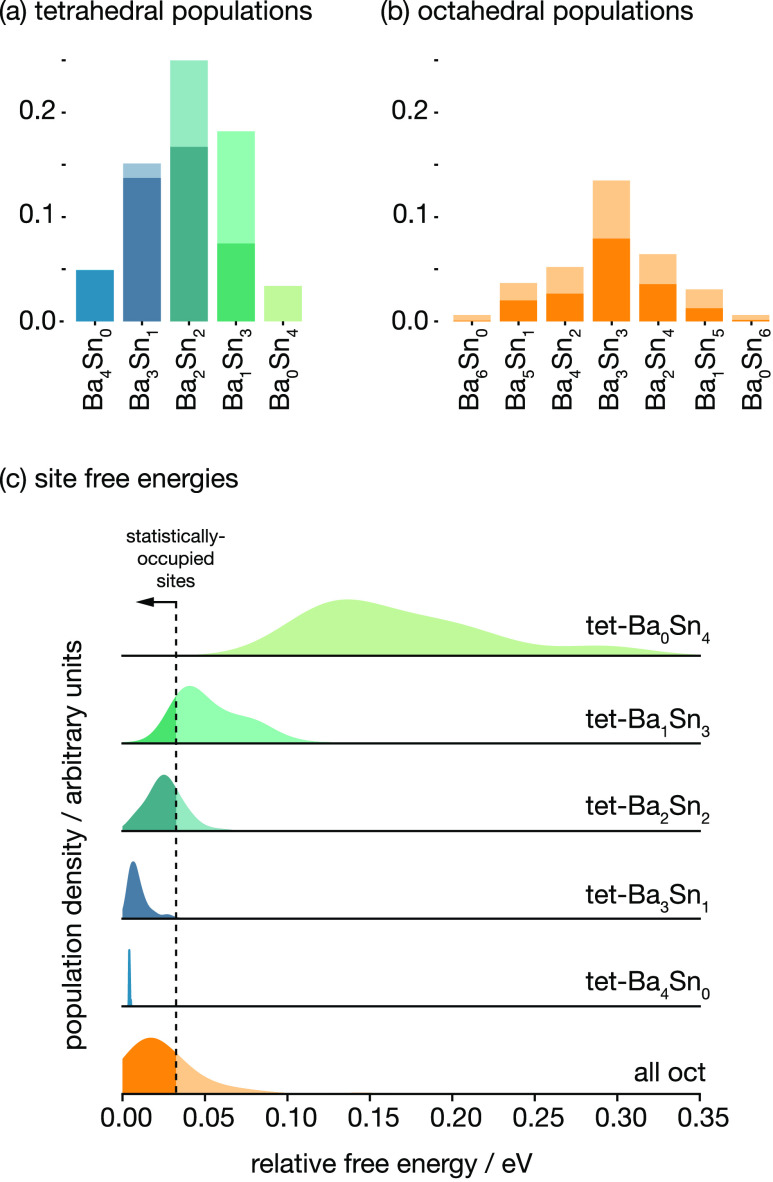
(a, b) (Lighter bars) Probability distribution (number frequency) of tetrahedral and octahedral sites, *p*(site), in the special quasi-random AIMD simulation cell, subclassified by the number of Ba and Sn that coordinate each site; (darker bars) joint probabilities for each site type being present in the simulation structure and being occupied by a fluoride ion, *p*(site ∩ occupied), calculated from AIMD simulation. The probability of a given site type being occupied by a fluoride ion, *p*(occupied | site), is given by the relative proportion of the darker bar to the lighter bar for each site type; *p*(occupied | site) = *p*(site ∩ occupied)/*p*(site). Numerical data are provided in a table in the Supporting Information. (c) Distributions of site-occupation relative free energies calculated from AIMD, grouped by fluoride-ion site type. Octahedral sites are shown as a single distribution.

Figure 7a,b shows the probability distribution (number frequency) of tetrahedral and octahedral sites in our AIMD simulation, subclassified by the number of Ba and Sn cations that coordinate each site. These figures also show the proportion of time during the simulation, or probability, that each type of site is occupied by a fluoride ion. For the tetrahedral sites, the occupation probability depends strongly on the identity of the coordinating cations: as the number of coordinating Sn increases, the probability of that site type being occupied by fluorine decreases. Comparing the limiting cases of exclusive Ba coordination and exclusive Sn coordination, Ba_4_-coordinated sites are occupied nearly 100% of the time, while Sn_4_-coordinated sites are nearly always vacant (raw numerical data are available in the Supporting Information). In contrast, for octahedral sites, the occupation probability depends much less strongly on the identity of the coordinating cations; each type of octahedral site is occupied approximately 2/3 of the time, although we do observe a weak preferential occupation of octahedral sites with equal numbers of coordinating Ba and Sn.

The probability of fluoride ions occupying a given site can be interpreted as the relative free energy for that site. Figure 7c shows distributions of per-site relative free energies calculated for each individual tetrahedral and octahedral site as Δ*F*_site_ = −*kT* ln(*P*_occ_), where *k* is the Boltzmann constant, *T* is the simulation temperature, and *P*_occ_ is the probability of each site being occupied, calculated from the AIMD simulation. These distributions can be thought of as effective “densities-of-states” of the different tetrahedral and octahedral site types. In Figure 7c, we also show a vertical line corresponding to the point where 2/3 of all available sites are statistically occupied, assuming that sites are preferentially occupied in order of increasing relative free energy.

In a conventional fluorite, the tetrahedral sites are low energy and the octahedral “interstitial” sites are higher energy. Moving an anion from a tetrahedral site to an octahedral site increases the total system energy, and forming Frenkel pairs is, therefore, a thermally activated process. Figure 7c illustrates how this conceptual model breaks down in c-BaSnF_4_, where the relative free energy of the tetrahedral sites increases with increasing Sn coordination. For sites with two or more coordinating Sn, some proportion of these sites are spontaneously depopulated, with the corresponding fluoride ions instead preferentially occupying octahedral sites. For Ba_1_Sn_3_- and Sn_4_-coordinated tetrahedral sites, this effect is large enough that these sites are nearly fully depopulated, contributing to the high octahedral site occupation. This behavior is consistent with a model where Sn lone pairs repel fluoride ions from adjacent tetrahedral sites, forcing these ions to instead occupy octahedral sites. The analysis presented here also indicates that this effect is additive; the more Sn cations coordinating a given tetrahedral site, the stronger the effective repulsion and the greater the bias to spontaneously depopulate these sites.

### Fluoride-Ion Dynamics

Having characterized the structure of c-BaSnF_4_, we now consider the fluoride-ion dynamics, and how this is affected by the structural features described above, first using ^19^F MAS NMR spectroscopy, and second by further analysis of our AIMD simulations.

**Figure 8:**
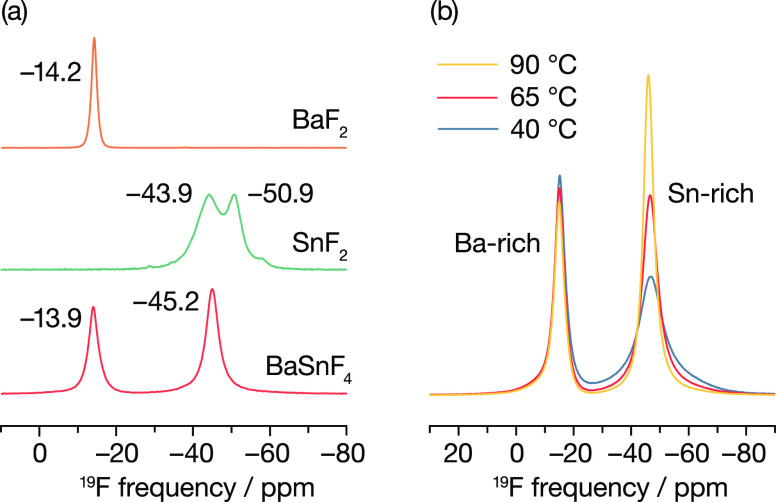
(a) ^19^F MAS (64 kHz) NMR spectra of c-BaSnF_4_ and its precursors BaF_2_ and α-SnF_2_. (b) Variable-temperature ^19^F MAS (30 kHz) of c-BaSnF_4_ recorded at 40, 65, and 90 °C. Fits to these c-BaSnF_4_ spectra are provided in the Supporting Information.

In M_x_^′^M_1–*x*_^″^F_2_ mixed-cation fluorites with no stereoactive lone pair, such as Ca_*x*_Ba_1–*x*_F_2_, ^19^F MAS NMR spectra show five distinct features corresponding to tetrahedral fluorine environments with different combinations of neighboring cation species, i.e., FM_4–*x*_^′^M_*x*_^″^ (*x* = {0, 1, 2, 3, 4}).^33,134,135^ The ^19^F MAS NMR spectrum for c-BaSnF_4_ instead shows only two distinct contributions at −14 ppm and −45 ppm (Figure 8a). The first of these peaks has a δ_iso_ value close to that of BaF_2_ (−14.2 ppm), where fluoride ions occupy Ba_4_-coordinated tetrahedral sites. The second peak aligns with the average δ_iso_ value of α-SnF_2_ (−46 ppm),^136^ in which fluorine is triply coordinated with short Sn–F distances.^137^ Based on these comparisons, we assign these features at −14 and −45 ppm to broadly Ba-rich and Sn-rich fluorine environments, respectively. The assignment of fluorine environments into broadly two types is qualitatively consistent with the computational fluoride-ion density data (Figure 6), where we observe quite different fluoride-ion densities in Ba-rich versus Sn-rich regions of our simulation model.

Our XRD data above indicate that Ba and Sn are randomly distributed across the fluorite 4a cation sites, and our AIMD simulations predict a complex fluorine substructure. Both results imply that c-BaSnF_4_ contains a rich variety of fluoride-ion environments, which might be expected to be observable in the experimental ^19^F MAS NMR spectrum, as in other mixed-cation fluorites;^33^ and yet we observe only two peaks. This apparent contradiction can be reconciled with our expectation of a complex fluorine substructure if we consider fluorine exchange between different sites within the host framework.^138^ Fluorine exchange between Ba-rich sites can cause individual peaks associated with different Ba-rich environments to coalesce, giving a single observed resonance. The same reasoning applies to Sn-rich environments, suggesting that they too exhibit fluorine exchange on the NMR time scale.

A third type of fluorine exchange is that between Ba-rich and Sn-rich environments, which we probe using variable-temperature ^19^F MAS NMR spectroscopy. Figure 8b shows spectra recorded at 40, 65, and 90 °C. As the temperature increases, the relative intensity of the peak assigned to fluoride ions in Sn-rich environments also increases, from 54 to 60%, at the expense of the peak assigned to fluoride ions in Ba-rich environments, confirming some degree of fluoride-ion exchange between Ba-rich and Sn-rich environments.

For a simple two-site exchange between Ba-rich and Sn-rich environments, increasing the temperature would be expected to produce a broadening of the associated resonances before their coalescence into a single resonance with an intermediate chemical shift. We do not observe such behavior, and instead the peaks assigned to Ba-rich and Sn-rich fluorine environments remain distinct across the investigated temperature range. This behavior is consistent with only some fraction of fluoride ions in Ba-rich environments undergoing exchange with ions in Sn-rich sites, with this fraction gradually increasing with temperature, and with this Ba-rich–Sn-rich exchange process being slower than the exchange between different Sn-rich environments;^138^ i.e., on the same time scale of exchange between Ba-rich and Sn-rich environments, fluoride ions in Sn-rich environments undergo exchange between several different Sn-rich environments.

The observation that fluoride-ion exchange between Sn-rich environments is much faster than that between Ba-rich environments or between Ba-rich and Sn-rich environments is further supported by the observation of motional narrowing of the Sn-rich peak with increasing temperature, indicating that the so-called fast-exchange regime is reached. This picture of locally inhomogeneous fluoride-ion dynamics is also qualitatively consistent with the time-average fluoride-ion density obtained from AIMD (Figure 6), where Ba-coordinated regions show highly localized fluoride-ion density, indicative of significantly less mobile ions, while Sn-coordinated regions show diffuse interconnected fluoride-ion density, suggesting more facile fluoride-ion motion between these sites.

To validate this model of faster fluoride-ion motion in Sn-rich regions, we performed additional analysis of our AIMD data to calculate site–site transition frequencies for each type of tetrahedral and octahedral sites. To estimate the degree to which these fluoride-ion site–site transitions contribute to long-range diffusion, rather than simple back-and-forth motion between adjacent sites, we also calculated frequencies of “nonreturning” transitions; these are transitions between two sites, 1 → 2, where the next transition made by the mobile ion takes it to a third site, 1 → 2 → 3, rather than returning it to the original site, 1 → 2 → 1.

**Figure 9:**
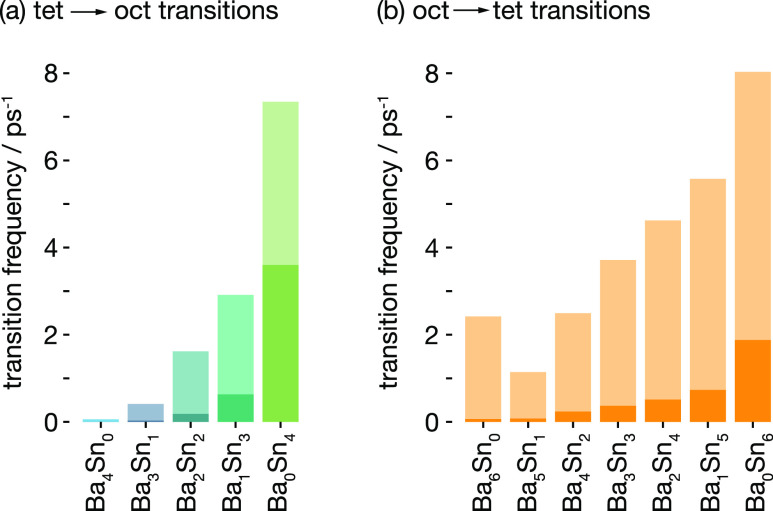
Site–site transition frequencies for fluoride ions in (a) tetrahedral and (b) octahedral sites, classified according to the cation nearest neighbors. Transition frequencies are normalized with respect to their time-average occupations, giving frequencies that are equivalent to inverse average site-occupation times. Lighter bars show data for all transitions and darker bars show data only for “nonreturning” transitions, as described in the main text.

The calculated site–site transition frequencies for tetrahedral and octahedral sites as a function of their Ba/Sn coordination are shown in Figure 9, normalized by the proportion of time each site type is occupied; this normalization gives transition frequencies that are equivalent to average inverse residence times; higher transition frequencies correspond to fluoride ions leaving a particular site more quickly. The calculated site–site transition frequencies for both tetrahedral and octahedral sites generally increase with increasing degree of Sn coordination, with this effect particularly strong for the tetrahedral sites. These data from AIMD simulation, therefore, are consistent with the model inferred from the variable-temperature NMR and fluoride-ion time-average density data (Figures 6 and 8b): fluoride ions in “Sn-rich” sites are, in general, more mobile than fluoride ions in “Ba-rich” sites.

### Sn Lone-Pair Dynamics

The ^19^F MAS NMR and AIMD data presented above show that the local mobility of fluoride ions in c-BaSnF_4_ is strongly dependent on the local cation composition: fluoride ions in Sn-rich environments are significantly more mobile than those in Ba-rich environments. An obvious partial explanation for this behavior is that the stereoactive lone pairs on tin cations somehow promote the motion of fluoride ions in adjacent tetrahedral and octahedral sites. Our calculated time-average fluoride-ion density (Figure 6) shows that the fluoride-ion substructure is highly diffuse in Sn-rich regions, which further suggests a possible direct interaction between the Sn lone pairs and the mobile fluoride ions.

**Figure 10:**
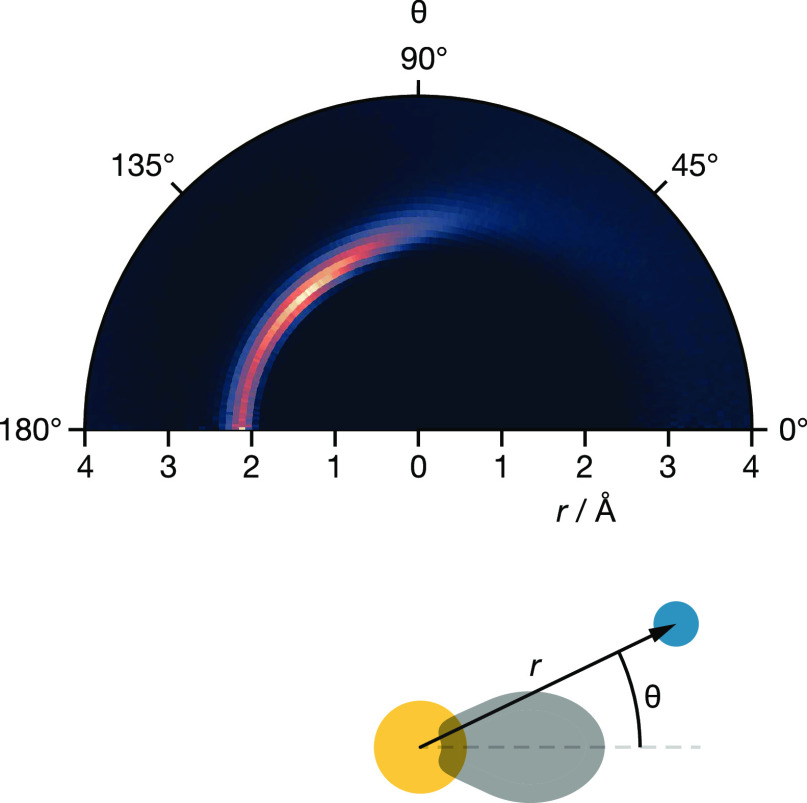
Sn–lone-pair–fluoride-ion polar spatial distribution function, *g*(*r*, θ), for c-BaSnF_4_, calculated from the AIMD simulation.

To quantify the degree of spatial correlation between the Sn lone pairs and nearby fluoride ions, we calculated the lone-pair–fluoride-ion polar spatial distribution function *g*(*r*, θ) (Figure 10). This distribution function describes the time-average fluoride-ion coordination environment around tin as a function of distance from the central tin cation, *r*, and the angle between the Sn–F vector and the lone-pair–orientation vector, θ. On the opposite side of the central tin from the lone pair, there is a clear feature at *r* = 2.1 Å with maximum intensity at 135°, i.e., the position of the tetrahedral 8c site if the lone pair is oriented toward the center of the opposite cube-face. On the lone-pair side, there is a distinct lack of structure and fluoride density is instead smeared out in a broad region from *r* > 3 Å. This distribution function is consistent with the model proposed from inspection of the time-average fluoride-ion density plot (Figure 6): the Sn lone pair is preferentially oriented toward one face of the enclosing cubic site, and fluoride ions that would occupy the corners of this face in a perfect fluorite structure are repelled by the lone pair, which strongly disrupts the fluoride structure in the vicinity of the lone pair.

**Figure 11:**
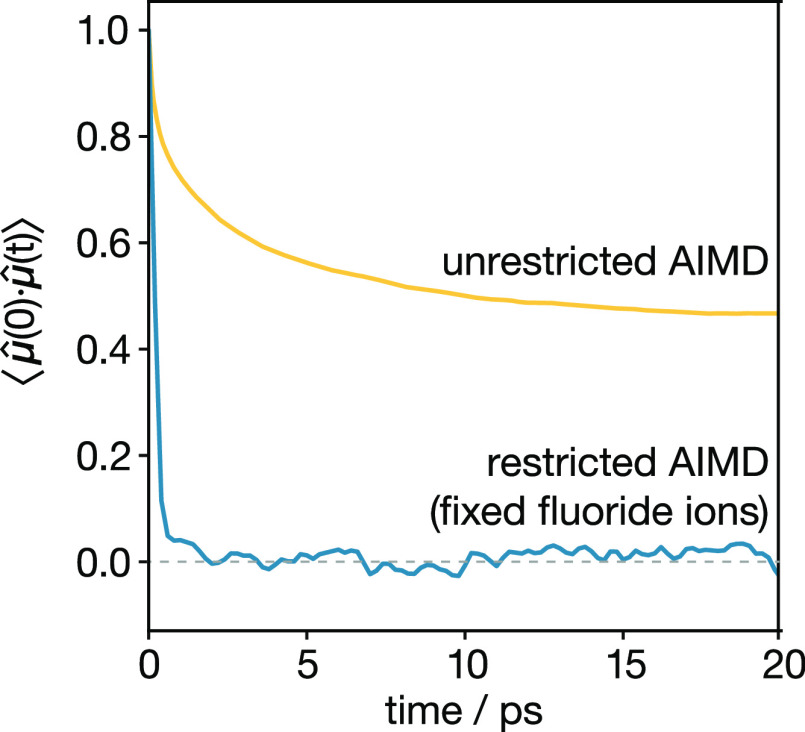
Sn-dipole orientational autocorrelation function for c-BaSnF_4_, for unrestricted AIMD, and for AIMD with fixed-position fluoride ions.

Another notable feature of the lone-pair fluoride-ion spatial distribution function is that the intense feature corresponding to fluoride ions occupying tetrahedral sites is angularly diffuse. While some of this effect can be attributed to the movement of these fluoride ions within their tetrahedral sites, it would be surprising for such movement to preserve the Sn–F separation. An alternative process that provides an explanation for the angular form of this feature is that the tin lone pair is reorienting relative to the reference fluorite lattice on a simulation time scale. To quantify any lone-pair reorientation dynamics, we calculated the Sn-dipole orientational autocorrelation function ⟨**μ̂**(0)·**μ̂**(*t*)⟩, which describes the average change in relative orientation of the stereoactive lone pairs in time *t*. This autocorrelation function (Figure 11) shows a clear decay on a picosecond time scale, showing that tin lone pairs in c-BaSnF_4_ undergo dynamic reorientation.

The Sn-dipole orientational autocorrelation function does not decay to zero. Therefore, on average, the orientation of each Sn lone pair is biased, with the lone pair more likely to point in one particular direction than in another. Plotting individual dipole-orientation autocorrelation functions for each lone pair (see the Supporting Information) shows that the strength of this bias varies significantly across tins, indicating that the degree of orientational bias is sensitive to the local tin environment.

To probe the degree to which the local tin environment directs the orientational bias for individual tin lone pairs, we calculated, for each lone pair, the proportion of time that this lone pair points toward each face of the enclosing cubic site. Each Sn has 12 cation nearest neighbors arranged in a cuboctahedron. For a given ⟨001⟩ vector from the central Sn, four of these cations are in front of the central Sn, and coordinate the fluoride sites on the front-face of the Sn 4a site, and four of these cations are behind the central Sn, and coordinate the fluoride sites on the back-face of the Sn 4a site; the other four neighboring cations occupy the same {001} plane as the central Sn. Because the local fluorine environment depends on the arrangement of the nearby Sn and Ba cations (as shown above; Figure 6), we consider the numbers of Ba and Sn cations coordinating the front-face and back-face of each tin as an effective descriptor for the degree to which a particular tin has a symmetric or asymmetric local coordination environment.

**Figure 12:**
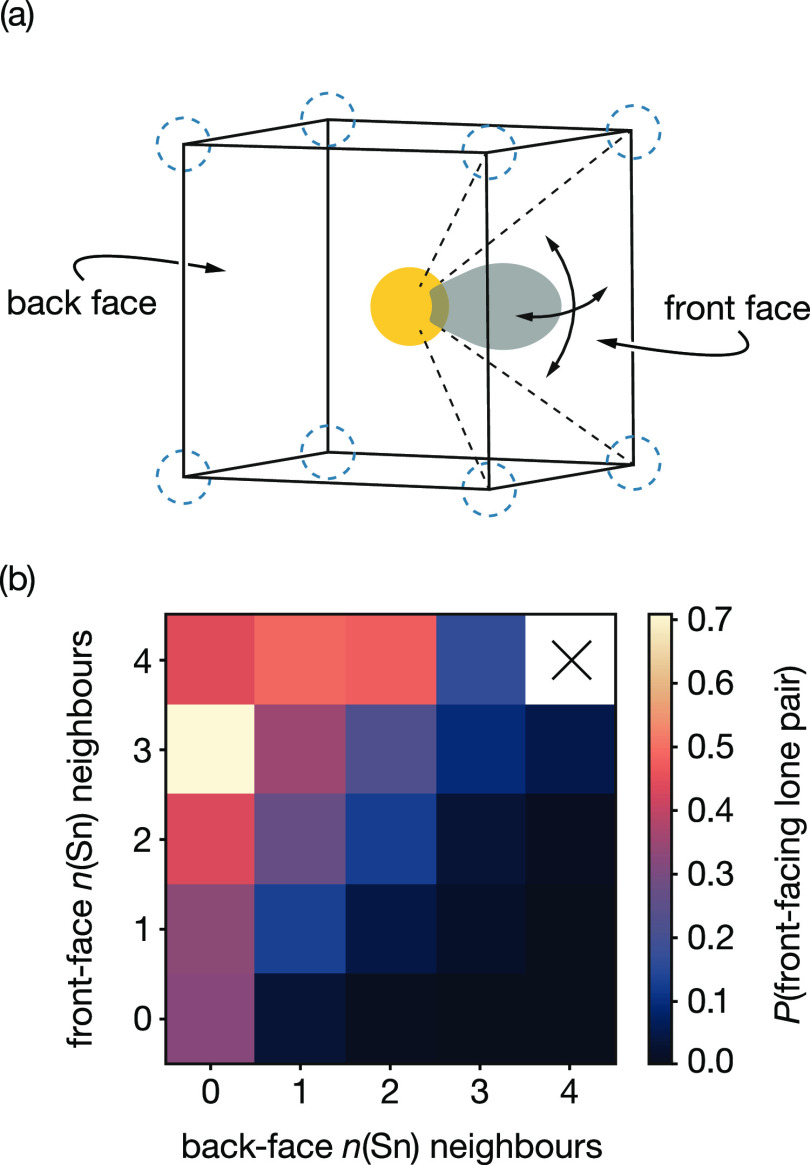
(a) Schematic of a Sn lone pair oriented toward one face of the cubic cation site. We consider a lone pair to be oriented toward a given face if the Sn dipole vector falls inside the square pyramid formed by the central Sn and the four corresponding vertex 8c positions. (b) Heat map for the proportion of time that a Sn lone pair is oriented toward a particular face of the cubic cation site, as a function of the number of nearest-neighbor tins in the {001} plane adjacent to the front-face of the cubic site (with respect to the lone-pair orientation) and the number of nearest-neighbor tins in the {001} plane adjacent to the back-face of the cubic site (with respect to the lone-pair orientation).

In Figure 12b, we show the proportion of time a lone pair points toward a given face of the enclosing cubic site, as a function of the number of nearest-neighbor Sn (out of a maximum of 4) that coordinate the front-face 8c sites and the number of nearest-neighbor Sn (again out of a maximum of 4) that coordinate the back-face 8c sites, with the data presented as a heat map. Data on the diagonal where *n*(Sn)_front_ = *n*(Sn)_back_ correspond to lone-pair orientations with symmetric front-face–back-face nearest-neighbor cation environments. These data all show relatively low values, indicating that lone-pair orientations with balanced cation coordination are weakly or negligibly biased. In contrast, lone pair orientations with more front-face Sn neighbors than back-face Sn neighbors show a strong bias. As a consequence, the stereoactive Sn lone pairs in c-BaSnF_4_, on average, tend to point toward other nearby tins. Clusters of Sn cations are therefore expected to have all of their lone pairs preferentially oriented toward the interior of the cluster, giving a cooperative effect where these Sn lone pairs all disrupt any fluoride-ion occupation of mutually coordinated tetrahedral sites. This model is consistent with the increasing tetrahedral site free energy with increasing Sn coordination (Figure 7) and provides an explanation for the extreme disruption of the fluoride substructure in Sn-rich regions, as observed in the fluoride-ion time-average density (Figure 6).

The time scale for lone-pair reorientation is similar to the time scale of fluoride-ion site–site transitions, which suggests possible coupling between these two kinds of dynamics. To examine whether the fluoride-ion dynamics and lone-pair dynamics are, in fact, coupled, we performed an additional AIMD simulation with all fluoride ions fixed at their ideal fluorite positions and calculated the corresponding Sn-dipole orientational autocorrelation function. With the fluoride ions fixed, the lone-pair orientational autocorrelation function decorrelates on a subpicosecond time scale (Figure 11), decaying to a rotationally symmetric (unbiased) value of zero.

This rapid decay of the Sn-dipole orientational autocorrelation function when the fluoride positions are fixed suggests that fluoride-ion dynamics and lone-pair dynamics are strongly coupled. When the fluoride ions are fixed to their 8c lattice positions, the lone pair moves freely; no matter which direction it points in, there is a strong lone-pair–fluoride repulsion. When the fluoride ions are free to move, however, a number of these fluoride ions move from unstable tetrahedral sites into more favorable octahedral sites, leaving vacant tetrahedral sites next to tin. The Sn lone pair preferentially orients toward these vacant sites to minimize the lone-pair–fluoride-ion repulsion (Figure 10). As fluoride ions move between sites, the Sn lone pairs dynamically reorient in concert with the changing local fluoride-ion configuration, giving strong coupling between the fluoride-ion dynamics and the lone-pair reorientation dynamics.

## Summary and Conclusions

To develop solid electrolytes with high ionic conductivities, it is necessary to understand how the chemistry of the host framework modulates the structure and dynamics of the mobile-ion species.^27,28,139,140^ In many families of solid electrolytes, introducing compositional or structural disorder within the host framework is an effective strategy for increasing ionic conductivity.^10,15,22−30^ (M,Sn)F_2_ fluorites have previously been proposed to exhibit two distinct forms of host-framework disorder:^62,88−90^ cation–site-occupation disorder, where the two cationic species are distributed randomly over the available sites, and Sn–lone-pair orientational disorder, where Sn exhibits stereoactive lone pairs with random orientations. This proposed coexistence of two distinct forms of host-framework disorder makes (M,Sn)F_2_ fluorites a particular focus of study in the context of understanding the possible interplay between disorder types and how, together, they modulate ion transport.

Here, we have investigated the structure and fluoride-ion dynamics of cation-disordered fluorite cubic (c-)BaSnF_4_. Rietveld refinement of XRD data confirms an average fluorite structure with {Ba,Sn} disorder (Figure 2). ^119^Sn Mössbauer spectroscopy demonstrates the presence of stereoactive Sn(II) lone pairs, and total-scattering PDF data show clear deviations from the average fluorite structure at short range (Figure 3).

Using *ab initio* molecular dynamics (AIMD) simulations, we have shown that the fluorine substructure in c-BaSnF_4_ is highly inhomogeneous and strongly dependent on the local cationic composition (Figures 4 and 6). In Ba-rich regions, the fluoride ions occupy fluorite-like tetrahedrally coordinated sites that form [F8] cubes around barium. In Sn-rich regions, in contrast, the fluoride-ion substructure is highly diffuse, with fluoride ions displaced from tetrahedral sites adjacent to Sn into octahedral “interstitial” sites.

We attribute the displacement of fluoride ions from tin-adjacent tetrahedral sites into octahedral interstitial sites to the presence of a stereoactive lone pair on the tin cations. The tin cations sit relatively close to their ideal fluorite positions and exhibit highly eccentric charge distributions that are characteristic of a stereoactive lone pair, in agreement with our ^119^Sn Mössbauer data. This lone-pair charge density destabilizes fluoride ions occupying adjacent tetrahedral sites, in effect pushing these fluoride ions into octahedral sites, thereby strongly disrupting the fluoride-ion substructure. This effect is clearly seen in the Sn-lone-pair–fluoride-ion polar spatial distribution function (Figure 10), where fluoride ions on the back-face of Sn sites—i.e., the opposite side from the lobe of the lone pair—are well structured, while fluoride ions on the front-face of Sn sites—in the direction the lone pair is oriented—are strongly repelled and highly disordered.

As a consequence of this Sn-lone-pair–fluoride-ion repulsion, c-BaSnF_4_ exhibits a remarkable concentration of “interstitial” fluoride ions that occupy octahedral sites. In our simulations, 1/3 of fluoride ions occupy octahedral sites, making it equally likely that, on average, octahedral sites and tetrahedral sites are occupied by fluoride ions. This level of octahedral site occupation exceeds that of previously reported “massively disordered” fluorites, such as RbBiF_4_,^132^ where 1/4 of fluoride ions occupy octahedral sites.^133^ In c-BaSnF_4_, this extreme fluoride-ion disorder is a consequence of a high relative free energy of occupation for tetrahedral sites adjacent to tin centers and an associated low relative free energy of occupation for octahedral sites (Figure 7), which is a consequence of the mutual repulsion between Sn-lone pairs and fluoride ions in adjacent tetrahedral sites.

We also directly probed fluoride-ion dynamics and the effect of cation disorder using variable-temperature ^19^F MAS NMR experiments and additional analysis of our AIMD data. Our NMR data show that fluoride ions in c-BaSnF_4_ can be broadly categorized as residing in either “Ba-rich” or “Sn-rich” environments, with fluoride ions in Sn-rich environments more mobile than fluoride ions in Ba-rich environments. This picture of cation-environment-dependent fluoride-ion dynamics is corroborated by our AIMD simulations: calculated site–site transition frequencies are higher for sites with a higher proportion of coordinating tin, showing a direct relationship between the local cation configuration and local anion dynamics.

Our AIMD simulations also reveal that the tin lone pairs dynamically reorient on a picosecond time scale (Figure 11). By comparing the results from our unrestricted AIMD simulations to equivalent data from simulations where the fluoride ions are fixed to their ideal fluorite positions, we have shown that the orientational dynamics of the tin lone pairs is coupled to the dynamics of the nearby fluoride ions. This effect is modulated by the local cation arrangement: for tins with an asymmetric Sn/Ba nearest-neighbor configuration, the tin lone pair preferentially orients in the direction of other, nearby, tins. Hence, clusters of tin cations exhibit a cooperative effect whereby the lone pairs on each tin tend to orient toward the interior of this cluster. This cooperative effect explains the dramatic disruption of the fluoride-ion substructure in regions where several Sn cations are clustered together (Figure 6).

The measured room-temperature ionic conductivity of c-BaSnF_4_ (4.6 × 10^–3^ S cm^–1^) is significantly higher than that of, for example, fluorite-structured BaF_2_,^24^ which is consistent with the general observation that within structurally related families of solid electrolytes, host-framework disorder is often correlated with increased ionic conductivities.^10,15,22−30^ In other materials, this relationship between host-framework disorder and ionic conductivity has been explained as a consequence of a concomitant disordering of the mobile-ion species that promotes ion transport,^10^ or of a reduction in differences in site-occupation energies between mobile-ion sites that flattens the mobile-ion potential energy surface.^15^ Our results for c-BaSnF_4_ are consistent with both of these conceptual models: in Sn-rich regions of the structure, the fluoride-ion density is highly diffuse (Figure 6), indicating significant local fluoride-ion disorder—which is also evident from our calculated F–F radial distribution function, Figure 5d—while our site-occupation analysis shows a destabilization of Sn-coordinated tetrahedral sites and a stabilization of octahedral sites that gives overlapping tetrahedral and octahedral site energies (Figure 7).

Given that c-BaSnF_4_ exhibits such a high degree of fluoride-ion disorder, it is perhaps surprising that it does not exhibit an even higher ionic conductivity. We observe greater fluoride-ion site disorder (1/3 of fluoride ions occupying octahedral sites) than in the mixed-valence mixed-cation fluorite RbBiF_4_ (1/4 of fluoride ions occupying octahedral sites), which would seem to predict a higher ionic conductivity for c-BaSnF_4_ than for RbBiF_4_. The room-temperature ionic conductivity of RbBiF_4_, however, is ×10^2^ greater than that of c-BaSnF_4_.^83^ This result can be explained by recognizing that fast-ion transport in solid electrolytes requires not only that there is a small, or nonexistent, energy gap between occupied and unoccupied sites, but also that these “frontier” sites form a contiguous percolating diffusion pathway through the material.^15^ In c-BaSnF_4_, the combined effects of cation disorder and lone-pair–fluoride-ion repulsion produce a large spread in tetrahedral site energies (Figure 7), causing tetrahedral sites with either high Ba coordination or high Sn coordination to be largely unavailable for long-range fluoride-ion diffusion. Highly Ba-coordinated tetrahedral sites (e.g., Ba_4_) have low site energies, are nearly fully occupied, and have low site–site transition frequencies, and fluoride ions occupying these sites are therefore largely immobile. As such, clusters of barium cations are expected to obstruct long-range fluoride-ion diffusion. Highly Sn-coordinated tetrahedral sites (e.g., Sn_4_) have a similar blocking effect on diffusion but for the opposite reason; these sites have high site energies and are therefore rarely occupied, despite having very high site–site transition frequencies. As a result, these Sn-coordinated sites obstruct long-range fluoride-ion diffusion by acting as high-energy bottlenecks. The remaining mixed-coordination tetrahedral sites (e.g., Ba_2_Sn_2_) then form a tortuous diffusion pathway, resulting in a lower macroscopic ionic conductivity than might be expected on the basis of local site–site transition frequencies or purely from the high level of fluoride-ion disorder present in the structure.

The results presented here demonstrate the complex interplay between two distinct forms of host-framework disorder (cationic site-occupation disorder and lone-pair orientational disorder) and the structure and dynamics of the mobile-ion species within a fluoride-ion-conducting solid electrolyte. The complex nature of these interacting effects suggests that the resulting effect on mobile-ion dynamics is likely to be highly dependent on the exact composition and structure of the solid electrolyte, and we expect further exploration of the coupling among crystallographic disorder, lone-pair dynamics, and ionic conductivity in solid electrolytes to be a fertile area for future research.

## Data Availability

A complete data set for the computational modeling and analysis described in this paper is available from the University of Bath Research Data Archive.^141^ This data set contains inputs and outputs for all DFT calculations, plus scripts for analysis of the DFT data and for plotting Figures 4–12b. A subsidiary data set containing only the figure-plotting scripts and relevant input data is available on GitHub.^142^

## Supporting Information Available

Full EDX analysis data; temperature-dependent ionic conductivity data; X-ray PDF data 12 Å ≤ *r* ≤ 50 Å; Mössbauer hyperfine parameters; comparisons between experimental PDF and simulated RDF data; fluoride-site and site-occupation probabilities; ^19^F MAS NMR data; and Sn-dipole orientational autocorrelation function data (PDF)
