# Supplementary material for: Dynamic Lone Pairs and Fluoride-Ion Disorder in Cubic-BaSnF4
Source: J Am Chem Soc. 2023 Oct 16;145(43):23739–54. doi: 10.1021/jacs.3c08232 (PMC10623577; doi:10.1021/jacs.3c08232)
Supplement: Supplementary file 1 — ja3c08232_si_001.pdf [file ja3c08232_si_001.pdf]

Dynamic Lone Pairs and Fluoride-ion Disorder in Cubic-$BaSnF_4$ (Supporting Information)

Briséïs Mercadier ,[1,2,3,*] Samuel W. Coles ,[4,5,*] Mathieu Duttine ,[6]
Christophe Legein ,[7] Monique Body ,[7] Olaf J. Borkiewicz ,[8] Oleg Lebedev,[9]
Benjamin J. Morgan ,[4,5,†] Christian Masquelier ,[1,3] and Damien Dambournet [1,2,‡]

[1]*Réseau sur le Stockage Electrochimique de l'Energie, RS2E, FR CNRS 3459, 80039 Amiens Cedex, France*
[2]*Sorbonne Université, CNRS, Physicochimie des Electrolytes et Nanosystèmes Interfaciaux, UMR CNRS 8234, 75005 Paris, France*
[3]*Laboratoire de Réactivité et de Chimie du Solides, UMR CNRS 7314, 80039 Amiens Cedex, France*
[4]*Department of Chemistry, University of Bath, Claverton Down BA2 7AY, United Kingdom*
[5]*The Faraday Institution, Quad One, Harwell Science and Innovation Campus, Didcot OX11 0RA, United Kingdom*
[6]*Institut de Chimie de la Matière Condensée de Bordeaux, UMR CNRS 5026, 33608 Pessac, France*
[7]*Institut des Molécules et Matériaux du Mans, UMR CNRS 6283, Le Mans Université, 72085 Le Mans Cedex 9, France*
[8]*X-ray Science Division, Advanced Photon Source, Argonne National Laboratory, Illinois 60439, United States*
[9]*Laboratoire de Cristallographie et Sciences des Matériaux, CRISMAT, 14000 Caen, France*

(Dated: October 14, 2023)

CONTENTS

S1. Full EDX analysis data 2

S2. Temperature-dependant ionic-conductivity data 2

S3. X-ray PDF data (12 Å $\leq r \leq$ 50 Å) 3

S4. Mössbauer hyperfine parameters 4

S5. Comparisons between experimental PDF and simulated RDF data 4

S6. Fluoride-site and site-occupation probabilities 4

S7. ^{19}F MAS NMR data 5

S8. Sn-dipole orientational autocorrelation function data 7

S1. FULL EDX ANALYSIS DATA

Fig. S1 shows EDX mapping of a particle of c$BaSnF_4$, showing a homogeneous dispersion of all three elements.

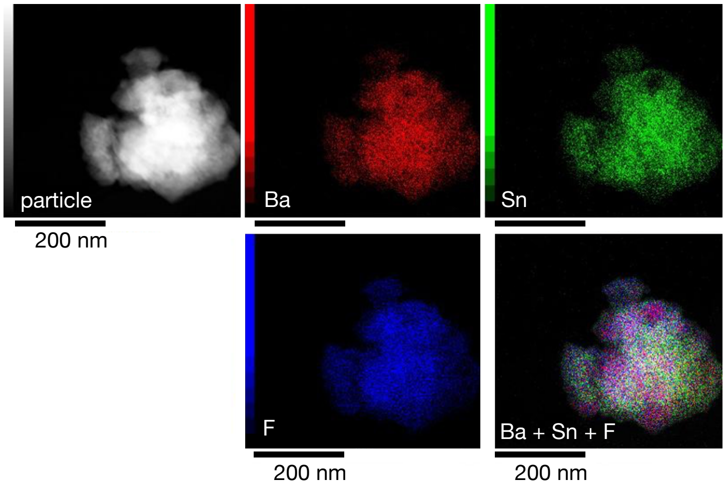

FIG. S1. EDX mapping of a particle of c-$BaSnF_4$.

S2. TEMPERATURE-DEPENDANT IONIC-CONDUCTIVITY DATA

Fig. S2 shows an Arrhenius plot of ionic conductivities for c-$BaSnF_4$ obtained from impedance spectroscopy and the Arrhenius model fitted to these data.

* These two authors contributed equally
† b.j.morgan@bath.ac.uk
‡ damien.dambournet@sorbonne-universite.fr

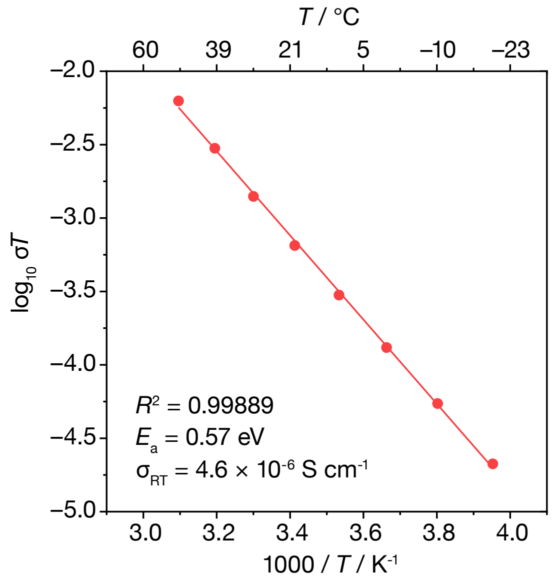

FIG. S2. Arrhenius plot of ionic conductivity as a function of temperature for c-$BaSnF_4$ for data from impedance spectroscopy, and the corresponding best-fit linear-least-squares Arrhenius model.

S3. X-RAY PDF DATA ($12\,\text{Å} \leq r \leq 50\,\text{Å}$)

Fig. S3 shows the experimental total-scattering pair distribution function (PDF) data for c-$BaSnF_4$ for the range $12\,\text{Å} \leq r \leq 50\,\text{Å}$ and the corresponding best-fit model PDF for a perfect fluorite structure.

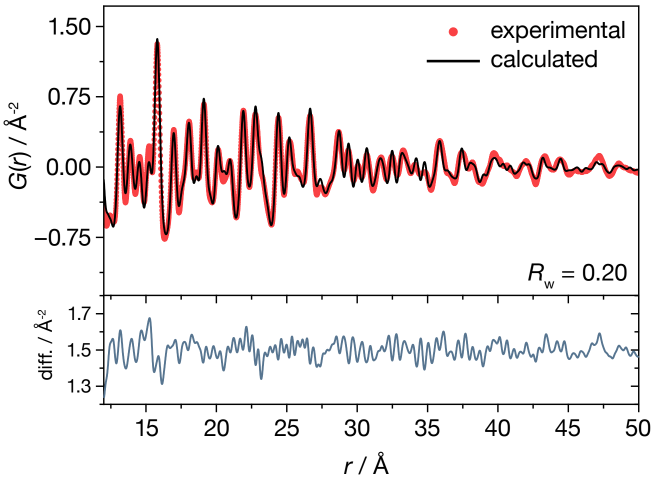

FIG. S3. PDF refinement obtained using a cubic model plotted for c-$BaSnF_4$ for the range $12\,\text{Å} \leq r \leq 50\,\text{Å}$.

S4. MÖSSBAUER HYPERFINE PARAMETERS

Table S1 reports the Mössbauer hyperfine parameters obtained by fitting the room-temperature (293 K) ^{119}Sn Mössbauer spectrum presented in the main text (Fig. 3(b)).

TABLE S1. Mössbauer hyperfine parameters for the c-$BaSnF_4$ room-temperature (293 K) ^{119}Sn Mössbauer spectrum presented in the main text (Fig. 3(b)). δ is the isomer shift relative to $CaSnO_3$ at room temperature, relative area describes the relative weight of the two contributions to the fitted spectrum, Δ is the quadrupole splitting, Γ is the Lorentzian line-splitting, and G_{11} is the Goldanskii–Karyagin factor.

samples	δ/mm s^{-1}	relative area	Δ/mm s^{-1}	Γ/mm s^{-1}	G_{11}
c-$BaSnF_4$ (contribution 1)	3.12(5)	59 %	1.77(5)	0.91(4)	1.27(4)
c-$BaSnF_4$ (contribution 2)	3.44(5)	39 %	1.55(6)	0.85(3)	1.26(4)

S5. COMPARISONS BETWEEN EXPERIMENTAL PDF AND SIMULATED RDF DATA

Figure S4(a) shows a visual comparison between the experimental PDF data for c-$BaSnF_4$ and the species-pairwise radial distribution functions (RDFs) calculated from our AIMD simulation. The simulated RDF data and experimental PDF data show excellent semi-quantiative agreement, with the RDF data reproducing well the M–F peak splitting at $\sim$ 2.0 Å to 2.6 Å and the M–M peak splitting at $\sim$ 4.0 Å to 4.6 Å. Figure S4(b) shows an equivalent comparison using RDFs calculated from a AIMD simulation where the fluoride ions were fixed at the Wyckoff $8c$ tetrahedral positions. The M–F and M–M peaks both fail to show the splitting seen in the experimental PDF data, with the nearest-neighbour M–F peak completely failing to describe the peak at 2.08 Å. This analysis provides further evidence for a significant degree of distortion to the fluorine substructure away from the reference simple-cubic structure.

S6. FLUORIDE-SITE AND SITE-OCCUPATION PROBABILITIES

Table S2 presents numerical data for site probabilities (number frequency), p(site), in the AIMD SQS structure, and for site-occupation probabilities, presented both as probabilities of observing a particular site type that is also occupied, $p(\text{site} \cap \text{occupied})$, and as probabilities that a given site type is occupied, normalised to the number of sites of each type present in the AIMD structure, $p(\text{occupied}|\text{site})$.

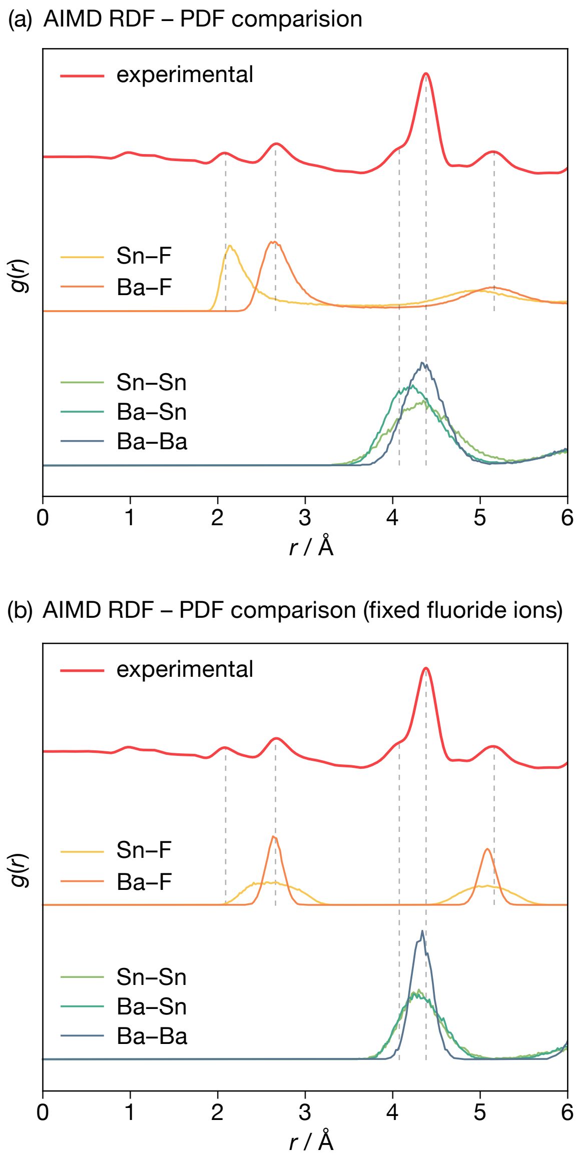

FIG. S4. (a) Comparison between the experimental PDF data for c-$BaSnF_4$ and the species-pairwise radial distribution functions (RDFs) calculated from our AIMD simulation. Vertical dashed lines show approximate positions of peaks in the experimental PDF data. (b) An equivalent comparison between the experimental PDF data for c-$BaSnF_4$ and RDFs calculated from AIMD simulation where the fluoride ions are fixed at the Wyckoff 8*c* positions.

S7. ^{19}F MAS NMR DATA

Fig. S5 shows the experimental ^{19}F MAS (64 kHz) NMR spectrum of c-$BaSnF_4$ (main text; Fig. 8), the corresponding fitted spectrum, and the individual resonances used in the fit. Parameters for the component resonances are listed in Table S3.

Fig. S6 shows equivalent experimental and fitted spectra for variable temperature ^{19}F (30 kHz) MAS NMR spectra of c-$BaSnF_4$, recorded at 40 °C, 65 °C, and 90 °C. Parameters

TABLE S2. Numerical data for site probabilities (number frequency), p(site), in the AIMD SQS structure, and for site-occupation probabilities, presented both as probabilities of observing a particular site type that is also occupied, $p(\text{site} \cap \text{occupied})$, and as probabilities that a given site type is occupied, normalised to the number of sites of each type present in the AIMD structure, $p(\text{occupied}|\text{site})$. The first two columns, p(site) and $p(\text{site} \cap \text{occupied})$, correspond to the light and dark bars, respectively, in Fig. 7(a) in the main text.

site type	p(site)	$p(\text{site} \cap \text{occupied})$	$p(\text{occupied}\|\text{site})$
tet Ba_4Sn_0	0.0494	0.0491	0.9939
tet Ba_3Sn_1	0.1512	0.1374	0.9084
tet Ba_2Sn_2	0.2500	0.1672	0.6688
tet Ba_1Sn_3	0.1821	0.0746	0.4099
tet Ba_0Sn_4	0.0340	0.0020	0.0599
oct Ba_6Sn_0	0.0062	0.0009	0.1483
oct Ba_5Sn_1	0.0370	0.0270	0.7283
oct Ba_4Sn_2	0.0525	0.0356	0.6786
oct Ba_3Sn_3	0.1358	0.1064	0.7831
oct Ba_2Sn_4	0.0648	0.0478	0.7375
oct Ba_1Sn_5	0.0309	0.0169	0.5464
oct Ba_0Sn_6	0.0062	0.0018	0.2950
Σ(site types)	1.0000	$0.666\bar{6}$	

TABLE S3. ^{19}F isotropic chemical shifts, δ_{iso} (ppm), full width at half-maximum, FWHM (ppm), relative intensities, I (%) of the NMR lines used for the reconstruction of the ^{19}F MAS (64 kHz) NMR spectra of c-$BaSnF_4$ (Fig. S5). The relative intensities, $\Sigma(I)$ and average isotropic chemical shifts, $\langle\delta_{\text{iso}}\rangle$, are reported for each peak.

δ_{iso}/ppm	I	FWHM	δ_{iso}/ppm	I	FWHM
−12.4	8.7	3.8	−42.3	4.7	9.1
−14.0	21.7	2.3	−45.0	47.5	3.6
−15.7	7.0	3.4	−47.6	9.5	8.5
$\langle\delta_{\text{iso}}\rangle = -13.9$ ppm			$\langle\delta_{\text{iso}}\rangle = -45.2$ ppm		
$\Sigma(I) = 37.8\,\%$			$\Sigma(I) = 62.2\,\%$		

for the component resonances are listed in Table S4.

For the 40 °C spectrum, a small additional peak was fit with a chemical shift of −31.5 ppm, a FWHM of 9 ppm and and a relative intensity of 1 %. Due to its small size and intermediate chemical shift, this fitted peak was not included as a contribution to either of the two main peaks.

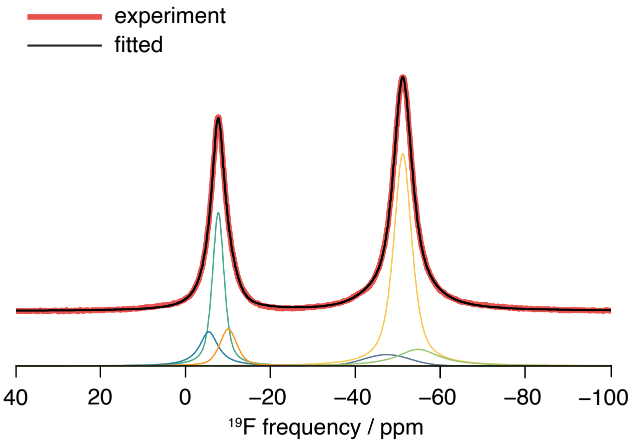

FIG. S5. Experimental and fitted ^{19}F MAS (64 kHz) NMR spectra of c-$BaSnF_4$. The lower section of the figure shows the individual resonances (see Table S3) used in the fit.

S8. SN-DIPOLE ORIENTATIONAL AUTOCORRELATION FUNCTION DATA

Fig S7 shows the AIMD-calculated Sn-dipole orientional autocorrelation functions for each tin in the $6 \times 6 \times 6$ AIMD supercell, illustrating the diverse range of long-time asymptotic values observed, indicating an environmental effect on the degree of orientational bias experienced by individual tin lone pairs.

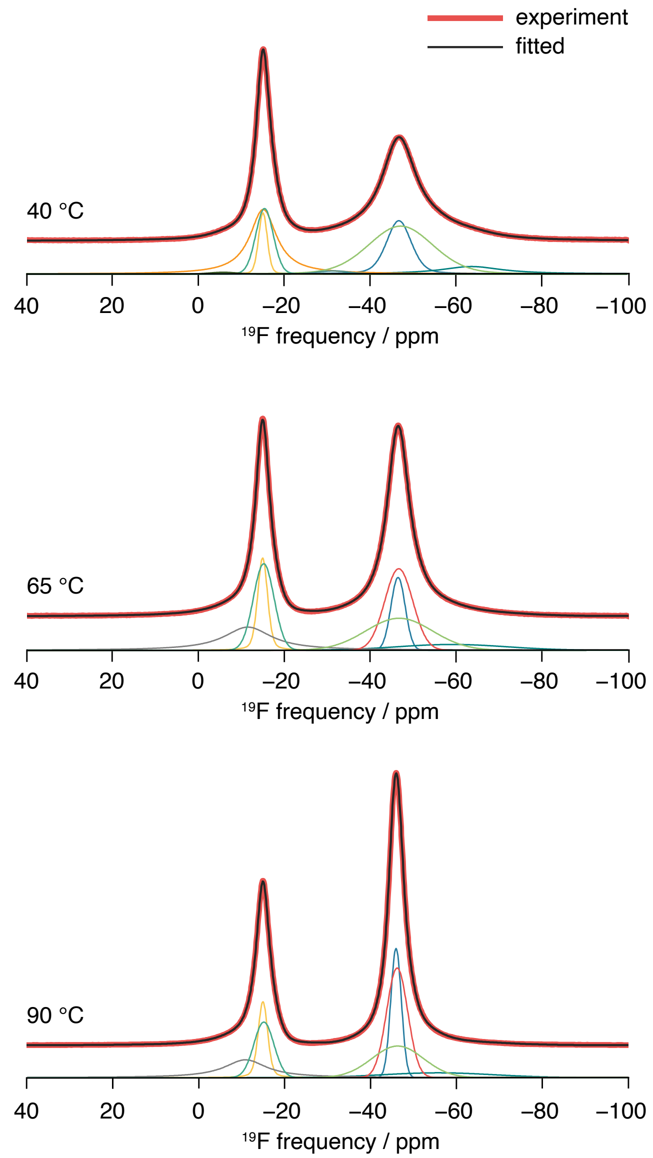

FIG. S6. Experimental and fitted ^{19}F MAS (30 kHz) NMR spectra of c-$BaSnF_4$, recorded at 40 °C, 65 °C, and 90 °C. The lower region of each plot shows the individual resonances used in each fit (see Table S4).

TABLE S4. ^{19}F isotropic chemical shifts, δ_{iso}, relative intensities, I, and full width at half-maximum, FWHM, of the NMR lines used to fit the ^{19}F MAS (30 kHz) NMR spectra of c-$BaSnF_4$ at 40 °C, 65 °C, and 90 °C (Fig. S6). The relative intensities, $\Sigma(I)$ and average isotropic chemical shifts, $\langle\delta_{iso}\rangle$, are reported for each peak.

40 °C

δ_{iso}/ppm	I	FWHM	δ_{iso}/ppm	I	FWHM
−15.5	11.1	4.8	−63.7	6.5	17.1
−15.2	28.2	7.5	−47.2	32.9	17.7
−15.1	5.5	2.4	−46.8	14.4	6.8
			−6	0.5	7.5
$\langle\delta_{iso}\rangle = -15.1$ ppm			$\langle\delta_{iso}\rangle = -49.1$ ppm		
$\Sigma(I) = 45.7\,\%$			$\Sigma(I) = 54.3\,\%$		

65 °C

δ_{iso}/ppm	I	FWHM	δ_{iso}/ppm	I	FWHM
−15.2	16.6	5.7	−59.1	5.7	30.0
−15.0	9.8	2.7	−46.8	19.2	18.3
−11.5	18.4	13.4	−46.7	21	7.7
			−46.5	9.4	3.9
$\langle\delta_{iso}\rangle = -13.7$ ppm			$\langle\delta_{iso}\rangle = -48.0$ ppm		
$\Sigma(I) = 44.8\,\%$			$\Sigma(I) = 55.2\,\%$		

90 °C

δ_{iso}/ppm	I	FWHM	δ_{iso}/ppm	I	FWHM
−15.2	17	5.5	−55.7	5.3	30.4
−15.0	8.5	2.6	−46.4	16.8	14.1
−10.9	14.2	11.9	−46.2	24.1	5.8
			−46.0	14.1	2.9
$\langle\delta_{iso}\rangle = -13.6$ ppm			$\langle\delta_{iso}\rangle = -47.0$ ppm		
$\Sigma(I) = 39.7\,\%$			$\Sigma(I) = 60.3\,\%$		

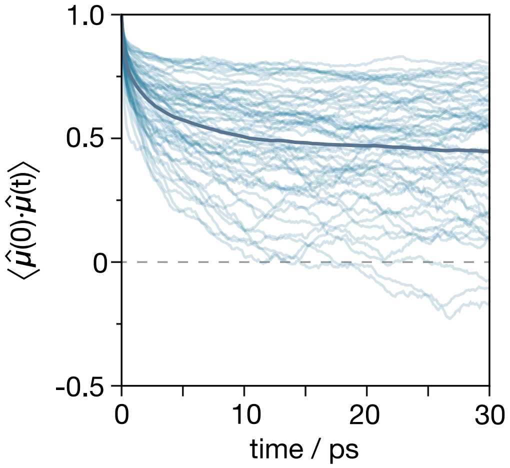

FIG. S7. Sn-dipole orientational autocorrelation functions for c-$BaSnF_4$ for each tin in the $6\times6\times6$ AIMD supercell. The bold line shows the average taken over all tins.
